# Increased IL-23R^+^ Th Cells Population Exhibits Higher SLEDAI-2K Scores in Systemic Lupus Erythematosus Patients

**DOI:** 10.3389/fimmu.2021.690908

**Published:** 2021-08-17

**Authors:** Aziz Farah Izati, Nur Diyana Mohd Shukri, Wan Syamimee Wan Ghazali, Che Maraina Che Hussin, Kah Keng Wong

**Affiliations:** ^1^Department of Immunology, School of Medical Sciences, Universiti Sains Malaysia, Kubang Kerian, Malaysia; ^2^Hospital Universiti Sains Malaysia, Kubang Kerian, Malaysia; ^3^Department of Internal Medicine, School of Medical Sciences, Universiti Sains Malaysia, Kubang Kerian, Malaysia

**Keywords:** systemic lupus erythematosus, IL-23/IL-17 axis, IL-17, IL-23, IL-17RA, IL-23R, SLEDAI-2K

## Abstract

The IL-23/IL-17 axis plays causative roles in the development and progression of systemic lupus erythematosus (SLE). However, it remains unclear if the IL-17RA^+^ and IL-23R^+^ T helper (Th) cells populations are associated with the serum IL-17 and IL-23 levels, or with the immunological parameters and disease activities in SLE patients. Herein, we examined the proportion of IL-17RA^+^ and IL-23R^+^ Th cells and serum levels of IL-17 and IL-23 in established SLE patients (n = 50) compared with healthy controls (n = 50). The associations of these interleukins and their receptors with immunological parameters [anti-nuclear antibody (ANA), anti-dsDNA antibody, and C-reactive protein (CRP)] and SLE disease activity (SLEDAI-2K scores) in SLE patients were assessed. CD3^+^CD4^+^ Th cells of SLE patients demonstrated significantly elevated IL-17RA^+^ (*p* = 1.12 x 10^-4^) or IL-23R^+^ (*p* = 1.98 x 10^-29^) populations compared with the healthy controls. Serum IL-17 levels were significantly lower in SLE patients compared with the healthy controls (*p* = 8.32 x 10^-5^), while no significant difference was observed for the IL-23 serum levels between both groups. IL-23R^+^ Th cells population was significantly associated with higher SLEDAI-2K scores (*p* = 0.017). In multivariate analysis, the proportion of IL-23R^+^ Th cells remained significantly associated with higher SLEDAI-2K scores independent of prednisolone intake (*p* = 0.027). No associations were observed between the interleukin parameters (i.e., IL-17, IL-23, IL-17RA^+^ Th cells, and IL-23R^+^ Th cells) with ANA, anti-dsDNA, and CRP status, suggesting that the IL-17/IL-23 axis acts independently of these immunological parameters. In conclusion, our results support that therapeutic inhibition of the IL-23/IL-17 axis receptors on Th cells, particularly IL-23R, is potentially relevant in SLE patients.

## Introduction

Interleukin (IL) is a group of cytokines crucial in regulating immune responses such as inflammation as well as mediating immune cells growth, differentiation, activation, and migration. Imbalanced T cell subsets have been identified in SLE patients associated with SLE immunopathogenesis (1–5). Naïve CD4^+^ T helper (Th) cells differentiate on activation into various cell subsets, namely Th1, Th2, and Th17, with specific profile of cytokines production and different immunopathological implications. IL-17 produced by Th17 cells has been implicated in the pathogenesis of autoimmune diseases including SLE (4). IL-23 is produced by macrophages and dendritic cells to promote the expansion and survival of Th17 cells for the production of IL-17 (6). By stimulating pro-inflammatory cytokines production, IL-23 is able to activate memory T cells, affect IFN-γ production, and trigger the response of Th1 cells, which plays crucial roles in the development of SLE (7–10). IL-23 can aggravate autoimmune severity by stimulating the activities of pathogenic Th17 cells to continually produce IL-17 (11), and this pathogenic pathway is termed as the IL-23/IL-17 axis in SLE (12, 13).

It is thought that cytokine receptors identification is more therapeutically beneficial on account of its stability as well as cytokines pleomorphism. Cytokine receptors activate intracellular signaling which controls a wide range of biological and clinically relevant functions including inflammatory reactions and the expansion of immune cells. The receptor for IL-17 cytokine, IL-17RA, has a cytoplasmic tail with docking sites for various signaling intermediates and has been recognized at elevated levels in specific tissues such as the thymus, bone marrow, spleen tissues, and hematopoietic cells (14). IL-23R, a receptor for IL-23, is expressed by various immune cells including dendritic cells, macrophages, and T cells (15). IL-23 and its receptor IL-23R are necessary for the proliferation of pathogenic IL-17-producing T cells shown to be critical for lupus pathogenesis (16), and IL-23R-deficit prevents the development of lupus nephritis in lupus-prone mice (16).

Multiple studies have reported that elevated IL-17 serum levels in SLE patients is associated with higher SLEDAI scores, a parameter to assess SLE disease activities (17–20), compared with the controls (21–26). Independent reports have indicated that Th17 cells can promote the development of SLE (22, 27). Although the initial steps of IL-17 differentiation from naïve CD4^+^ Th cells does not involve IL-23, it has been shown that IL-23 plays a significant role in the expansion and maintenance of Th17 cells, enabling IL-17 to be produced (7, 28–30). Collectively, the IL-23/IL-17 axis plays key roles in the pathogenesis of SLE. However, the profile of IL-17RA^+^ or IL-23R^+^ Th cells in SLE patients compared with healthy individuals, as well as their associations with relevant parameters in the disease, remain unclear.

In this study, we set out to investigate whether serum IL-17 and IL-23 levels or the proportion of IL-17RA^+^ or IL-23R^+^ Th cells differ between SLE patients and the healthy controls. More importantly, the potential associations between these serum cytokines and Th cell subsets with SLE disease activity measured by the SLEDAI-2K scores were examined. Associations of the SLEDAI-2K scores with the clinico-demographical (e.g., age) and immunological (e.g., ANA, anti-dsDNA) parameters were also investigated, and compared with the IL-23/IL-17 axis parameters using multivariate and false discovery rate (FDR) analyses.

## Materials and Methods

### Recruitment of SLE Patients and Healthy Controls

According to published flow cytometry and/or ELISA case-control studies utilizing peripheral blood samples to investigate IL-17 or IL-23 parameters in SLE patients compared with controls (31–35), sample size calculation was performed according to the difference of means between two independent groups using the software G*Power (version 3.1.9.3). A two-tailed hypothesis, α-error probability of 0.05, power (1-β error probability) of 0.80, effect size of 0.6, allocation ratio (N2/N1) of 1, and dropout rate of 10% were adopted. This yielded a total sample size of 100 participants divided equally between the healthy controls and SLE patients group (n = 50 per group). Thus, in this cross-sectional case-control study, 100 participants were recruited consisting of 50 established SLE patients who fulfilled the 2012 Systemic Lupus International Collaborating Clinics (SLICC) criteria (36) for SLE diagnosed from the year 2012 onwards, or the 1997 Update of the 1982 American College of Rheumatology Revised Criteria for Classification of SLE (37) for patients diagnosed before 2012, and 50 healthy control volunteers. Subject recruitment was conducted based on the inclusion and exclusion criteria (**Supplementary Table 1**). The selection of SLE patients and healthy controls for this study was conducted on the basis of random sampling. The clinico-demographic data of the patients were obtained from the Records Unit of Hospital Universiti Sains Malaysia (HUSM). SLEDAI-2K score for each patient was assigned by the attending clinician at the time of this study (i.e., on the same day when the samples were taken) according to the SLEDAI-2K score calculator (https://qxmd.com/calculate/calculator_335/sledai-2k) (38). Data on the current treatments received (i.e., prednisolone, immunosuppressants, or anticonvulsant) were obtained at the time of the study. Recruitment of the subjects and retrieval of their data were conducted from November 2018 to May 2019. The study procedures were approved by the Human Research Ethics Committee of Universiti Sains Malaysia (JEPeM) (approved ethics code: USM/JEPeM/17120680). All procedures carried out in this study involving peripheral blood samples were in accordance with the 1964 Declaration of Helsinki and its later updates, and with the institutional ethical standards. All participants were briefed about the study during the recruitment, and both verbal and written informed consents were obtained from each participant after detailed explanations of the study. The documented written informed consent permitted the investigators to conduct the recruitment, draw the blood of the participants for the experiments, and publish the results. All samples were labeled anonymously, and all data were recorded, stored, and analyzed anonymously to ensure that none of the private information such as the name of participants were disclosed. All experimental protocols were conducted according to the institutional relevant guidelines and regulations.

### Serum and PBMCs Isolation

A total of 10 mL of peripheral blood was withdrawn from the healthy controls (n = 50) and SLE patients (n = 50). Of this, 6 mL were allocated in a clot activator tube for serum collection and 4 mL was placed in a BD Vacutainer^®^ EDTA tube (Becton Dickinson, USA) for peripheral blood mononuclear cells (PBMCs) isolation. The collected blood samples were left in an upright position for 30 minutes at room temperature (RT) prior to centrifugation at 1,000 x g at 4°C for 15 min to remove the blood clot. The serum obtained from centrifugation was aliquoted immediately into 1.5 ml microcentrifuge tubes which were labeled with the identification number of the subject and the intended tests (e.g., ANA, anti-dsDNA, CRP, IL-17, IL-23), and stored at -20°C. On the other hand, PBMCs were isolated from peripheral blood by standard Histopaque-1077 (Sigma-Aldrich, USA) density centrifugation. The collected peripheral blood was inverted and left in an upright position for 30 min at RT. Four milliliters of Histopaque-1077 was transferred into a new falcon tube followed by 4 mL of peripheral blood from the EDTA tube layered carefully on top of the Histopaque-1077 with a 1:1 ratio. The blood mixture was then centrifuged at 400 x g (30 min at RT, brakes off). The PBMCs layer obtained was transferred carefully using a Pasteur pipette into a new falcon tube containing 2 mL of PBS. The mixture was then added with PBS to a total of 10 mL, mixed gently, and resuspended. The supernatant obtained after being centrifuged at 200 x g for 10 min was discarded and the pellet was resuspended using 5 mL of PBS and centrifuged. The washing process was then repeated with 500 μl of PBS before cell counting using a hemocytometer.

### Flow Cytometry Analysis for IL-17RA^+^ and IL-23R^+^ Th Cells

A total of 5 mL of isolated PBMCs were centrifuged at 1,000 rpm for 10 min. The cells were washed with 1 ml of cold FBS followed by centrifuging at 1,000 rpm for 15 min. Then, cells were resuspended with cold FBS to a final concentration of 2 x 10^6^ cells/ml. The cell mixtures were distributed and 50 μl of cell suspension was aliquoted into a microcentrifuge tube. Fluorochrome-labeled antibodies for CD3 (APC-Cy7; cat no. 560176), CD4 (PE-Cy7; cat no. 557852), IL-17RA (BB700/PerCP-Cy5.5; cat no. 747946), IL-23R (FITC; cat no. FAB14001F), and isotype controls (APC-Cy7; cat no. 560167, PE-Cy7; cat no. 557872, BB700/PerCP-Cy5.5; cat no. 566404, FITC; cat no. GZ-IC0041F) (Beckton Dickinson, USA) were used at 5 μl per test whereby the antibody concentration for each test was 0.0182 μg/μl in a final volume of 55 μl, corresponding to 1 μg of antibody per test according to the protocols of the manufacturer. Each antibody was added accordingly into the cells mixture and incubated on ice protected from light for 30 min. Washing steps were conducted twice with 1 ml of FBS, the tube inverted, and supernatant blotted from the cell pellets. The cell pellets were resuspended in 500 μl of FBS. The stained cells were then transferred into an FACS tube and analyzed using the FACS CANTO II (Beckton Dickinson). The results were acquired with the FACS Diva software (Beckton Dickinson) and analyzed with FlowJo (v10; TreeStar, USA).

### ELISA for Serum IL-17 and IL-23 Levels

ELISA tests were conducted according to manufacturer’s instructions (BioLegend, USA). Briefly, monoclonal antibodies specific for human IL-17 (cat no. 433917) and IL-23 (cat no. 437607) were pre-coated onto the provided 96-well strip microplate. Standards and samples for both IL-17 and IL-23 levels detection were run in duplicate, and the standard curve was obtained for each assay. All reagents were brought to RT prior to use, and 50 ml of 20X wash buffer was diluted in 950 ml of sterilized dH_2_O to prepare a 1X wash buffer. Each microplate used was labeled accordingly, and the washing process to remove unbound materials was conducted with 300 μl of 1X wash buffer per well, and the residual buffer was blotted by tapping firmly the microplate upside down on a clean absorbent paper. The final washing process was conducted for 30 seconds to 1 min for each wash to minimize the background. Absorbance values were recorded at 450 nm and 570 nm by the ELISA microplate reader (Tecan, Switzerland).

### Anti-Nuclear Antibodies Indirect Immunofluorescence Assay (IIFA)

The IIFA for the AESKUSLIDES ANA-HEp-2 test was performed according to the instructions of the manufacturer (AESKU DIAGNOSTICS, Germany). All components of AESKUSLIDES-ANA-Hep-2 were allowed to reach RT before usage. Serum samples were diluted following the ratio of 1:80 with PBS (20 μl of serum + 1,580 μl of PBS), and 25 μl of each diluted serum and controls (ready-to-use format) were pipetted into the appropriate wells. Slides were incubated in a humidified chamber for 30 min at RT. The slides were subsequently rinsed with the 1X wash buffer using a squeeze wash bottle followed by 10 min of washing in a glass staining dish containing fresh wash buffer. The slides were then transferred to the humidified chamber and excess wash buffer was carefully removed by filter paper. Subsequent incubation with secondary antibody conjugated with FITC was conducted in the humidified chamber for 30 min at RT in the dark. Washing steps were then repeated before a coverslip was mounted on each slide. The specific green fluorescent staining of the antigen-antibody complex was observed and interpreted with an Olympus BX-60 fluorescence microscope (Olympus, Tokyo, Japan). Interpretation of the ANA staining patterns were conducted by immunologists (KKW, CMCH) according to the International Consensus on ANA Patterns (ICAP; http://www.anapatterns.org/) nomenclature and definitions of 29 HEp-2 IIFA patterns, where each pattern is ascribed with an alphanumeric code (AC-1 to AC-29) (39).

### Anti-dsDNA ELISA Test

ImmuLisa™ Anti-dsDNA Antibody Enhanced ELISA tests were conducted according to the instructions of the manufacturer (Immco Diagnostics, USA). Anti-dsDNA IgG antibodies were detected by the test. Firstly, the microwells were coated with purified dsDNA antigen followed by blocking steps to reduce non-specific binding. Controls, calibrators, and patient sera were incubated in the antigen-coated wells, allowing the specific antibody in the serum to bind to the dsDNA antigens. Bound antibodies were detected by adding the anti-human IgG conjugate enzyme to the microwells and the presence of the antibodies were detected by a color change produced after a specific enzyme substrate (TMB) were added. After the washing process, to remove the unbound antibody and conjugate, the reaction was stopped and the intensity of the color change was detected by a spectrometer at 450 nm. Linear measurement was recorded and the results were reported as anti-dsDNA antibody positive or negative.

### C-Reactive Protein Immunoturbidimetric Test

QuikRead go CRP test (Orion Diagnostica, Finland) is an immunoturbidimetric test based on microparticles coated with anti-human CRP F(ab)_2_ fragments, and the test was carried out according to manufacturer’s instructions. The CRP present in the serum reacts with the microparticles resulting in the turbidity of the solution as quantitatively measured by the QuikRead go instrument.

### Statistical Analysis

The categorical and numerical data for the clinico-demographical and immunological characteristics of SLE patients and healthy controls were described using frequency (%) and median (interquartile rage; IQR), respectively. Normality of data was tested using the Shapiro-Wilk test. For comparison of numerical variables between two groups, unpaired t-test or Mann-Whitney test was used for normally and not normally distributed variables, respectively. Kruskal-Wallis test and the subsequent Dunn’s multiple comparisons *post hoc* test were used to compare numerical variables in more than two groups. For correlation analysis between two sets of numerical variables, Pearson and Spearman correlation coefficient was used for normally and not normally distributed variables, respectively. For categorical variables, their associations were compared using the Chi-square test and Fisher’s exact test (used when >20% of cells have expected frequencies below five) (40). Statistical analysis was performed using the GraphPad Prism (v9; GraphPad Software Inc., USA). Multivariate analysis of IL-23R^+^ Th cells combined with age or prednisolone intake *versus* SLEDAI-2K score groups (i.e., SLEDAI-2K score 0 or ≥1) was conducted using multiple logistic regression (IBM SPSS v22; SPSS Inc., Chicago, IL, USA). For all tests, two-tailed *p*<0.05 was considered statistically significant.

## Results

### Clinico-Demographical Characteristics of the Participants

A total of 50 SLE established patients and 50 healthy controls were recruited in this study. The SLE patients and healthy controls were mostly female (98% and 82%, respectively). Majority of the SLE patients were ethnic Malay (n = 46/50; 92%) followed by non-Malay (n = 4/50; 8%), and the same was observed for the healthy controls with 82% (n = 41/50) Malay and 18% (n = 9/50) non-Malay. In our SLE cohort, patients aged 31–40 years were the most common, followed by 21–30 years and other age groups (**Supplementary Figure 1**). A number of SLE patients (n = 13/50; 26%) had a family history of autoimmune diseases while none in the healthy controls group. Among the SLE patients and healthy controls enrolled in this study (n = 100), two healthy controls were smokers (n = 2/50; 4%) and all SLE patients were non-smokers. The SLEDAI-2K scores of SLE patients were calculated and recorded with a median score of 1.50 (IQR: 0–6.50). In terms of medications, 90% (n = 45/50) of SLE patients took hydroxychloroquine, followed by corticosteroid (n = 37/50; 76%), other immunosuppressants (n = 21/50; 42%), and anticonvulsant (n = 1/50; 2%) (**Table 1**).

**Table 1:** Clinico-demographical and immunological characteristics of SLE patients and healthy controls.

Characteristics^a^	SLE patients (n = 50)	Healthy Controls (n = 50)
**Median age (years) (IQR)**	34.0 (26.0–40.8)	26.5 (24.0–32.0)
**Gender**		
** Female**	49 (98.0)	41 (82.0)
** Male**	1 (2.0)	9 (18.0)
**Ethnicity**		
** Malay**	46 (92.0)	41 (82.0)
** Non-Malay**	4 (8.0)	9 (18.0)
**Family history of autoimmune disease**		
** Yes**	13 (26.0)	0 (0.0)
** No**	37 (74.0)	50 (100.0)
**Smoking status**		
** Yes**	0 (0.0)	2 (4.0)
** No**	50 (100.0)	48 (96.0)
**SLEDAI-2K (median) (IQR)**	1.50 (0–6.50)	NA
**Use of medicines**		
** Corticosteroids**		
** Prednisolone**	38 (76.0)	NA
** Immunosuppressant**		
** Azathioprine**	20 (40.0)	NA
** Hydroxychloroquine**	45 (90.0)	NA
** Methotrexate**	1 (2.0)	NA
** Anticonvulsant**		
** Sodium valproate**	1 (2.0)	NA
**Anti-nuclear antibody (ANA)**		
** Positive**	45 (90.0)	NA
** Negative**	5 (10.0)	NA
**Anti-dsDNA antibody, (n = 50)**		
** Positive (≥50 IU/ml)**	36 (72.0)	NA
** Negative (<50 IU/ml)**	14 (28.0)	NA
**C-reactive protein (CRP)**		
** Normal (<3 mg/l)**	28 (56.0)	NA
** Mildly elevated (3 ≤ x < 10 mg/l)**	16 (32.0)	NA
** Elevated (≥10 mg/l)**	6 (12.0)	NA

a Data are presented as number (percentage) unless stated otherwise.

### Immunological Characteristics of SLE Patients

**Figure 1:**
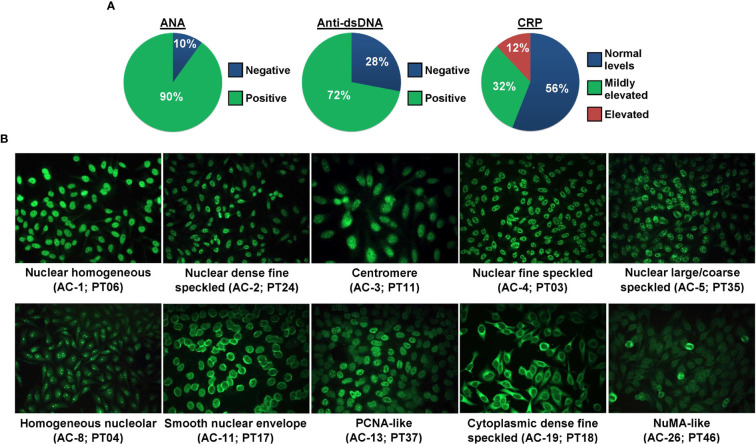
ANA, anti-dsDNA, and CRP status of SLE patients. **(A)** The proportion of SLE patients (n = 50) positive or negative for ANA and anti-dsDNA, and negative, mildly elevated, or elevated for CRP. **(B)** ANA patterns of SLE patients. Ten ANA patterns were observed in our cohort of SLE patients and each pattern is shown by a representative case. The ICAP HEp-2 IIFA pattern in alphanumeric code and the patient ID are stated in brackets.

The patients had their SLE diagnosis established previously where all patients were positive for ANA, and 36 out of 43 (83.7%; anti-dsDNA test was not initially conducted for 7 patients) were positive for anti-dsDNA antibody leading to their SLE diagnosis according to the established criteria. In the current study, ANA and anti-dsDNA tests were conducted anew along with the CRP test. The proportion of SLE patients according to their ANA (n = 45/50 ANA-positive; 90%), anti-dsDNA (n = 36/50 anti-dsDNA-positive; 72%), or CRP status is presented in **Figure 1A**. The proportion of SLE patients positive for ANA (n = 45/50; 90%) was higher than those negative for ANA (n = 5/50; 10%, i.e., ANA seroconversion to ANA-negative post-treatment). The ANA and anti-dsDNA test results of SLE patients prior to disease diagnosis and current ANA and anti-dsDNA results as well as CRP levels are summarized in **Supplementary Table 2**.

Ten distinct ANA patterns implicated in the SLE diagnosis were observed in our cohort of SLE patients where most of the cases displayed a nuclear large/coarse speckled pattern (AC-5; n = 19/45; 42.2%), followed by nuclear homogeneous (AC-1; n = 7/45; 15.6%), nuclear fine speckled (AC-4; n = 7/45; 15.6%), smooth nuclear envelope (AC-11; n = 5/45; 11.1%), homogeneous nucleolar (AC-8; n = 2/45; 4.4%), and one case each (2.2%) for nuclear dense fine speckled (AC-2), centromere (AC-3), PCNA-like (AC-13), cytoplasmic dense fine speckled (AC-19), and NuMA-like (AC-26). Representative fluorescent images for each of these 10 ANA patterns are shown in **Figure 1B**, and fluorescent images for the rest of the ANA-positive and ANA-negative cases are shown in **Supplementary Figure 2**. The detailed ANA pattern interpretations for each SLE case are described in **Supplementary Table 3**. The proportions of the different ANA patterns observed in our cohort of cases are comparable with those of published studies utilizing the ICAP nomenclature (**Supplementary Table 4**). A total of 68.9% (n = 31/45) of the ANA-positive SLE patients were also positive for the anti-dsDNA antibody. Majority of the SLE patients had normal CRP levels (n = 28; 56%) compared with patients presented with mildly elevated (n = 16; 32%) or elevated (n = 6; 12%) CRP levels (**Table 1**).

### Determination of IL-17RA^+^ and IL-23R^+^ Th Cells Populations by Flow Cytometry

The peak of flow cytometry histogram for unstained lymphocytes were fixed at 1 for all fluorochromes (PE-Cy7, APC-Cy7, PerCP, and FITC). To observe a distinct peak between the negative and positive cells populations, the histograms of isotype control (red shaded) and fluorochrome-stained (blue shaded) were overlapped for each fluorochrome to distinguish the negative and positive cells populations (**Supplementary Figure 3**). The gating strategy for CD3^+^CD4^+^ Th cells and subsequent determination of IL-17RA^+^ or IL-23R^+^ Th cells subset is summarized in **Supplementary Figure 4**.

### IL-17RA^+^ and IL-23R^+^ Th Cells Populations in SLE Patients *Versus* Healthy Controls

**Figure 2:**
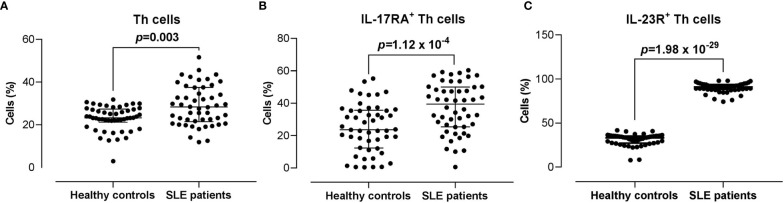
Proportion of CD3^+^CD4^+^ Th cells, and IL-17RA^+^ and IL-23R^+^ Th cells subsets in SLE patients *versus* healthy controls. **(A)** Proportion of CD3^+^CD4^+^ Th cells in SLE patients (n = 50) *vs* healthy controls (n = 50). **(B, C)** Proportion of IL-17RA^+^ **(B)** and IL-23R^+^ **(C)** Th cells in SLE patients *vs* healthy controls. The bars in each scatter plot represent the median and interquartile range.

The proportion of CD3^+^CD4^+^ Th cells in SLE patients was significantly higher compared with the healthy controls (28.30% *vs* 23.35%) (*p* = 0.003). Moreover, the proportions of IL-17RA^+^ (39.45% *vs* 23.65%; *p* = 1.12 x 10^-4^) and IL-23R^+^ (90.95% *vs* 33.25%; *p* = 1.98 x 10^-29^) Th cells were significantly higher in SLE patients compared with the healthy controls (**Figure 2**).

By taking into account only Malay subjects (SLE n = 46 *vs* healthy controls n = 41), the proportions of IL-17RA^+^ (39.45% *vs* 22.90%; *p* = 2 x 10^-4^) and IL-23R^+^ (90.95% vs 33.20%; *p* = 1.75 x 10^-25^) Th cells remained significantly higher in SLE patients compared with the healthy controls (**Supplementary Figure 5**).

### Serum IL-17 and IL-23 Levels in SLE Patients and Healthy Controls

**Figure 3:**
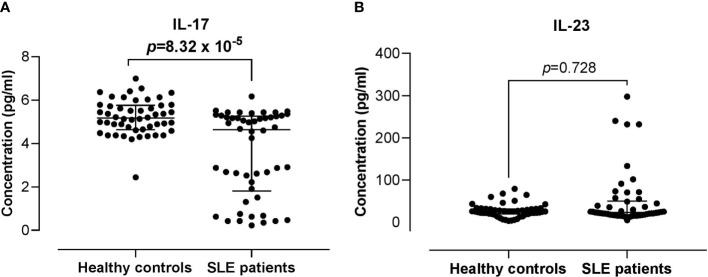
Comparison of the serum IL-17 and IL-23 levels between SLE patients *versus* healthy controls. **(A)** IL-17 serum levels in SLE patients *vs* healthy controls. **(B)** IL-23 serum levels in SLE patients *vs* healthy controls. The bars in each scatter plot represent the median and interquartile range.

**Figure 4:**
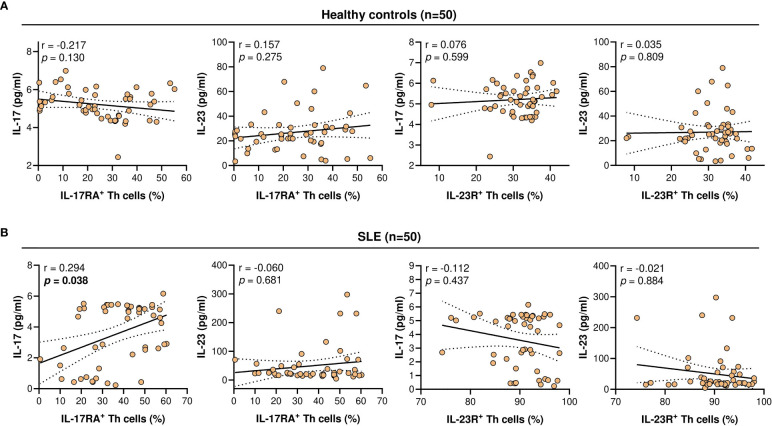
Correlation of IL-17RA^+^ or IL-23R^+^ Th cells population (%) with serum IL-17 or IL-23 levels (pg/ml). **(A)** Correlation in healthy controls (n = 50). **(B)** Correlation in SLE patients (n = 50). For each graph, the linear regression plot is represented by a best-fit line surrounded by adjacent 95% confidence bands.

Serum IL-17 levels were significantly lower in SLE patients compared with the healthy controls (4.63 *vs* 5.16 pg/ml; *p* = 8.32 x 10^-5^). The serum levels of IL-23 did not differ significantly between the SLE patients and healthy controls (22.85 *vs* 25.23 pg/ml; *p* = 0.728) (**Figure 3**). Higher serum IL-17 levels were significantly and positively associated with the proportion of IL-17RA^+^ Th cells in SLE patients (r = 0.294; *p* = 0.038). However, these associations were not observed in the healthy controls. In addition, serum IL-17 and IL-23 levels were not associated with the proportion of IL-23R^+^ Th cells in both the healthy controls and SLE patients (**Figure 4**).

### Higher Proportion of IL-23R^+^ Th Cells, Younger Age, and Prednisolone Intake Were Associated With Higher SLEDAI-2K Scores

Next, we assessed the association of the clinico-demographical and immunological characteristics with the SLEDAI-2K scores, i.e., SLEDAI-2K score 0 (n = 23) *vs* ≥1 (n = 27) in SLE patients. Younger SLE patients (36 *vs* 30 years old; *p* = 0.042), prednisolone intake (56.5% *vs* 88.9%; *p* = 0.009), and higher proportion of IL-23R^+^ Th cells (89.10% *vs* 92.40%; *p* = 0.017) were significantly associated with higher SLEDAI-2K scores (**Table 2**). The rest of the parameters that were examined did not exhibit such significance with the SLEDAI-2K scores, i.e., ethnicity (*p* = 0.614), history of autoimmune disease (*p* = 0.191), immunosuppressant intake (*p* = 0.441), ANA-positive (*p* = 0.651), anti-dsDNA-positive (*p* = 0.324), CRP status (*p* > 0.999), serum IL-17 levels (*p* = 0.508), serum IL-23 levels (*p* = 0.466), and the proportion of IL-17RA^+^ Th cells (*p* = 0.743) (**Table 2**).

**Table 2:** Association of the clinico-demographical and immunological characteristics with SLEDAI-2K scores in SLE patients (n = 50).

Characteristics		SLEDAI-2K		*p*-value
		0 (n = 23)	≥1 (n = 27)	
**Age (years) (median; IQR)**		36.0 (31.0–49.0)	30.0 (24.0–38.0)	**0.042** ^a^
**Ethnicity (n; %)**	Malay	22 (95.7)	24 (88.9)	0.614^b^
	Chinese	1 (4.3)	3 (11.1)	
**History of autoimmune disease (n; %)**	Yes	8 (34.8)	5 (18.5)	0.191^c^
	No	15 (65.2)	22 (81.5)	
**Prednisolone (n; %)**	Yes	13 (56.5)	24 (88.9)	**0.009** ^c^
	No	10 (43.5)	3 (11.1)	
**Immunosuppressant (n; %)**	Yes	11 (47.8)	10 (37.0)	0.441^c^
	No	12 (52.2)	17 (63.0)	
**ANA (n; %)**	Yes	20 (87.0)	25 (92.6)	0.651^b^
	No	3 (13.0)	2 (7.4)	
**Anti-dsDNA (n; %)**	Yes	15 (65.2)	21 (77.8)	0.324^c^
	No	8 (34.8)	6 (22.2)	
**CRP (mg/ml) (n; %)**	<3	13 (56.5)	15 (55.6)	>0.999^b^
	3≤ x <10	7 (30.5)	9 (33.3)	
	≥10	3 (13.0)	3 (11.1)	
**IL-17 (pg/ml) (median; IQR)**		4.56 (1.91–5.17)	4.67 (1.51–5.28)	0.508^a^
**IL-23 (pg/ml) (median; IQR)**		22.7 (16.43–35.55)	23.01 (18.90–57.0)	0.466^a^
**IL-17RA^+^ Th cells (%) (median; IQR)**		41.80 (25.40–50.20)	36.20 (25.20–47.60)	0.743^d^
**IL-23R^+^ Th cells (%) (median; IQR)**		89.10 (87.30–91.70)	92.40 (89.20–95.0)	**0.017** ^a^

a Mann-Whitney test.

b Fisher’s exact test.

c Chi-square test.

d Unpaired t-test.

p < 0.05 in bold.

In addition, we also examined the association between serum IL-17 and IL-23 levels, and IL-17RA^+^ or IL-23R^+^ Th cells population with the immunological characteristics in SLE patients. No significant association was observed in any of the comparisons made between serum IL-17 or IL-23 levels, and IL-17RA^+^ or IL-23R^+^ Th cells proportion with ANA, anti-dsDNA, or CRP status (**Supplementary Table 5**).

### Multivariate and False Discovery Rate Analyses of the Associations With SLEDAI-2K Scores

**Figure 5:**
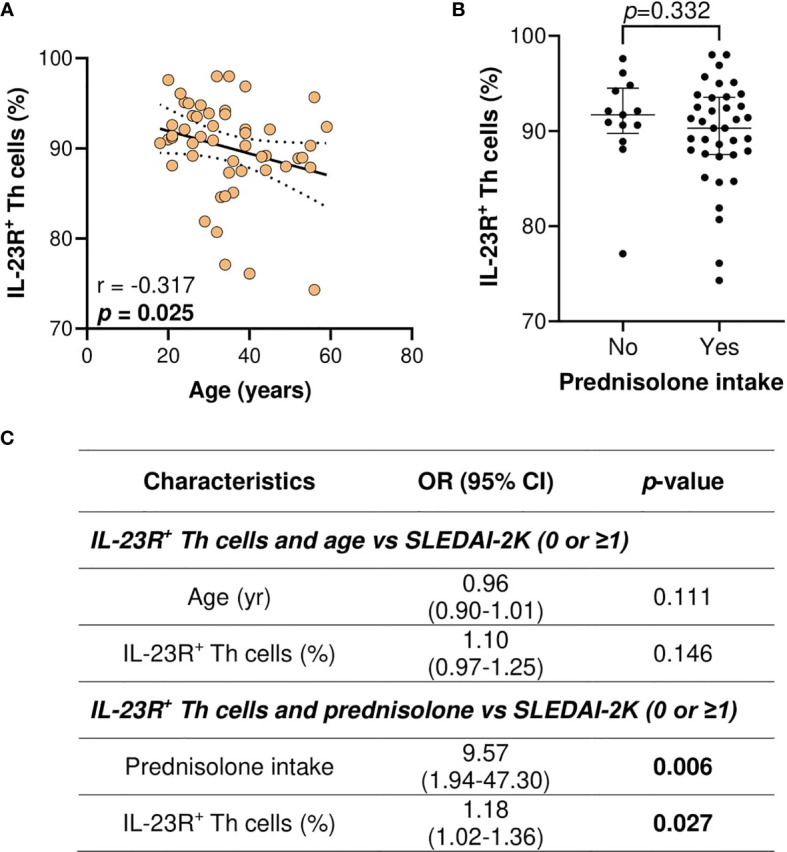
Association of IL-23R^+^ Th cells (%), age (years), and prednisolone intake with the SLEDAI-2K scores in SLE patients. **(A)** Association of IL-23R^+^ Th cells with age in SLE patients. **(B)** Association of IL-23R^+^ Th cells with prednisolone intake in SLE patients. **(C)** Multivariate analysis of IL-23R^+^ Th cells and age or prednisolone intake *vs* SLEDAI-2K (0 or ≥1) in SLE patients (n = 50).

As younger age, prednisolone intake, and the proportion of IL-23R^+^ Th cells were associated with higher SLEDAI-2K scores, the univariate association of IL-23R^+^ Th cells proportion with age or prednisolone intake was first assessed. Higher IL-23R^+^ Th cells proportion was significantly associated with a younger age (*p* = 0.025) (**Figure 5A**), and no significant association was observed for prednisolone intake (**Figure 5B**). Multivariate analysis of IL-23R^+^ Th cells and age or prednisolone intake *versus* SLEDAI-2K scores demonstrated that IL-23R^+^ Th cells proportion was not significantly associated with higher SLEDAI-2K scores when age was taken into account (OR: 1.10, 95% CI: 0.97–1.25; *p* = 0.146). However, IL-23R^+^ Th cells proportion remained significantly associated with higher SLEDAI-2K scores when prednisolone intake was taken into account (OR: 1.18, 95% CI: 1.02–1.36; *p* = 0.027) (**Figure 5C**).

A total of 36 associations were tested for a potential significance in this study where 6 of the associations demonstrated significant *p*-values. To account for multiple hypothesis testing, the *p*-values of these associations were corrected using the Benjamini-Hochberg (BH) FDR procedure. The threshold to use for significance for the generated *q*-values (BH-corrected *p*-values) was 0.25 (41, 42). Two of the six significant associations were rendered insignificant through BH correction, i.e., serum IL-17 *vs* IL-17RA^+^ Th cells in SLE patients (*q* = 0.252), and age *vs* SLEDAI-2K scores (*q* = 0.252). The rest of the four associations remained significant, i.e., IL-23R^+^ Th cells *vs* SLEDAI-2K scores (*q* = 0.204), prednisolone intake *vs* SLEDAI-2K scores (*q* = 0.162), multivariate analysis of prednisolone intake (tested with IL-23R^+^ Th cells combination) *vs* SLEDAI-2K scores (*q* = 0.162), and multivariate analysis of IL-23R^+^ Th cells (tested with prednisolone intake combination) *vs* SLEDAI-2K scores (*q* = 0.243) (**Supplementary Table 6**). Finally, the complete data of the clinico-demographical and immunological characteristics, as well as the serum interleukin and their receptors data for each individual healthy control and SLE patient involved in this study, are presented in **Supplementary Table 7**.

## Discussion

In this study, we demonstrated that CD3^+^CD4^+^ Th cells were significantly higher in SLE patients compared with the healthy controls. In terms of IL-23/IL-17 axis receptors, we observed that IL-17RA^+^ and IL-23R^+^ Th cells were significantly increased in SLE patients than in the healthy controls. This is comparable with another study where it was reported that the percentages of CD4^+^IL-23R^+^ cells from PBMCs was significantly higher in SLE patients (35). The production of IL-17 is driven by IL-23 (11, 43) and IL-23R is expressed on activated Th17 cells (44). Furthermore, the increased IL-17RA^+^ and IL-23R^+^ Th cells populations in SLE patients may be attributable to their shared molecular signaling pathway as the binding of IL-23 with its receptor complex activates STAT3 signaling in Th17 cells leading to IL-17 production through differentiation of Th17 cells (45, 46). Along with our observation of increased IL-23R^+^ Th cells in SLE patients associated with higher SLEDAI-2K scores, IL-23R^+^ Th cells may play crucial roles in the progression and aggressive disease course of SLE patients.

Multiple studies have reported significantly elevated serum IL-23 levels in SLE patients compared with healthy controls (12, 33, 47). Specifically, serum IL-23 levels were significantly higher in active SLE patients compared with inactive and healthy controls group (32, 48). However, our study demonstrated that serum IL-23 levels did not differ significantly between SLE patients and healthy controls, suggesting that increased IL-23R signaling might be achieved *via* higher surface levels of IL-23R instead of serum IL-23 levels. On the other hand, serum IL-17 was lower in SLE patients but IL-17RA^+^ Th cells population was higher in the patients, suggesting that a higher consumption of serum IL-17 by increased IL-17RA may occur in SLE patients. This is also supported by our observation that serum IL-17 levels had a positive relationship with IL-17RA^+^ Th cells in SLE patients but not in the healthy controls. However, these hypotheses require further validation by functional and mechanistic studies such as gene knockdown or knockout methodologies.

In addition, significantly downregulated serum IL-17 levels in our cohort of SLE patients is in contrast with the findings of elevated serum IL-17 levels in SLE patients as reported previously (22–24, 26). This may also be due to the use of hydroxychloroquine, an immunosuppressant routinely used to treat SLE patients (27, 49–52), by the majority of our cohort of SLE patients (n = 45/50; 90%). It has been reported that hydroxychloroquine use resulted in a significant reduction of disease activities in SLE patients where most of the cytokines and pro-inflammatory markers were reduced following the treatment (51–54). Hydroxychloroquine has been shown to inhibit Th17 cells proliferation and differentiation along with reduced IL-17 production by Th17 cells *in vitro* (27). Essentially, SLE patients (n = 6) demonstrated lower levels of serum IL-17 and reduced proportion of CD3^+^CD8^-^IL-17^+^ Th17 cells after receiving 4 weeks of treatment with hydroxychloroquine and prednisone (27). Furthermore, in PBMCs derived from a cohort of 18 SLE patients, stimulation of these PBMCs with phorbol 12-myristate 13-acetate and ionomycin displayed reduced IL-17 production after treatment with hydroxychloroquine (55). Elevated serum levels of IL-17 correlated positively with the SLEDAI-2K scores in SLE patients as reported in previous studies (26, 56). In contrast, several studies demonstrated that there were no associations observed between IL-17 (21, 57) or IL-23 (58) with the SLEDAI-2K scores, similar with the findings in our study.

In our multivariate analysis, IL-23R^+^ Th cells and SLEDAI-2K association was dependent on age in our SLE cohort, tallying with the univariate comparison where younger SLE patients had a significantly higher proportion of IL-23R^+^ Th cells. These observations are comparable with independent studies in which younger SLE patients are more likely to present with nephritis and greater organ damage (59–62), and more likely to be hospitalized with SLE as a diagnosis (59). Whether an increased proportion IL-23R^+^ Th cells plays a direct, causative role in exacerbating disease activities in younger SLE patients remains to be elucidated. On the other hand, IL-23R^+^ Th cells proportion remained significantly predictive of worse SLEDAI-2K scores when prednisolone intake was taken into account in the multivariate analysis, suggesting that prednisolone did not affect IL-23R^+^ Th cells proportion in SLE patients. This also suggests that alternative therapeutics specifically targeting IL-23R or its downstream signaling may be beneficial for patients who fail to respond to prednisolone.

In terms of immunological parameters, 10% of the established SLE patients in our cohort demonstrated ANA seroconversion. Loss of ANA over time in established SLE patients has been reported in up to approximately 30% of the patients (63–67). ANA seroconversion may reflect responses to therapies or naturally-occurring exhaustion of autoimmune clones (66), and whether our population of SLE patients displayed a reduced retention of autoimmune clones post-therapy is subject to future investigations. We observed that the routine biomarker anti-dsDNA did not correlate with the SLEDAI-2K scores in SLE patients. This is in agreement with multiple independent reports whereby anti-dsDNA status does not show significant relationship with SLE disease activities (68–70). As anti-dsDNA antibodies do not always correlate with SLE disease activities, it is thought that anti-dsDNA encompasses a large spectrum of different chromatin targets that may explain their heterogeneity in lupus activities (70, 71). This proposal appears to tally with past observation in which IgA, but not IgG or IgM, anti-dsDNA was significantly elevated in SLE patients with higher disease activities (72). This represents fertile grounds for further investigation and validation. In terms of CRP, it has been reported that IL-17 correlates with CRP levels and other immunology diagnostic parameters including IgG and IgM (57). However, our study demonstrated that there were no associations between the interleukin parameters (i.e., IL-17, IL-23, IL-17RA^+^ Th cells, and IL-23R^+^ Th cells) with the immunological parameters investigated in this study, suggesting that the IL-17/IL-23 axis acts independently of ANA, anti-dsDNA, and CRP.

We acknowledge the limitations of the study as follows: 1) Established SLE patients were recruited whereby all patients had been on treatment, hence, a proportion of the patients demonstrated ANA seroconversion and lower disease activities; 2) A confirmatory cohort of SLE patients especially of SLE patients recruited from multiple centers is recommended to validate and expand on the findings of the current study; and 3) We did not investigate which Th cell subsets expressing IL-17RA or IL-23R were altered such as Th1 and Th17 populations. Nonetheless, we recommend this as an avenue for future investigations particularly for IL-23R.

In conclusion, our study demonstrated that increased populations of IL-17RA^+^ and IL-23R^+^ Th cells occurred in SLE patients, and IL-23R^+^ Th cells population was associated with higher SLEDAI-2K scores. Our studies support future research either in the production or clinical trials of therapeutic antibodies targeting IL-23/IL-17 axis receptors in SLE, particularly IL-23R, and the inhibition of IL-23/IL-17 receptors is a promising therapeutic strategy in this autoimmune disorder.

## Data Availability Statement

The datasets presented in this study can be found in online repositories. The names of the repository/repositories and accession number(s) can be found in the article’s/**Supplementary Material**.

## Ethics Statement

The studies involving human participants were reviewed and approved by Human Research Ethics Committee of Universiti Sains Malaysia (JEPeM) (approved ethics code: USM/JEPeM/17120680). All patients and participants provided their written informed consent to participate in this study.

## Author Contributions

CMCH and KKW conceived the study and recruited research grants. AFI, NDMS, WSWG and CMCH recruited the samples and retrieved the clinico-demographical data of the subjects. AFI and NDMS conducted the experiments, and KKW supervised the experiments. AFI, CMCH and KKW designed the study. AFI and KKW performed data analysis, generated the figures and tables, conducted literature searches, and wrote the manuscript. KKW revised the manuscript. All authors contributed to the article and approved the submitted version.

## Funding

This research project was supported by the Research University grant (1001/PPSP/8012246) by Universiti Sains Malaysia awarded to CMCH and the Research University grant (1001/PPSP/8012349) awarded to KKW.

## Conflict of Interest

The authors declare that the research was conducted in the absence of any commercial or financial relationships that could be construed as a potential conflict of interest.

## Publisher’s Note

All claims expressed in this article are solely those of the authors and do not necessarily represent those of their affiliated organizations, or those of the publisher, the editors and the reviewers. Any product that may be evaluated in this article, or claim that may be made by its manufacturer, is not guaranteed or endorsed by the publisher.

## Supplementary Material

Supplementary Figure 1 Distribution of age in 50 SLE patients.

Supplementary Figure 2 ANA patterns of the rest of the SLE patients (n = 40). The ICAP HEp-2 IIFA pattern definition with alphanumeric code and patient ID are stated above and below, respectively, the fluorescent images.

Supplementary Figure 3 The histogram of isotype control (red shaded) and single color stained (blue shaded) were overlapped to determine negative and positive populations; PE-Cy7 **(A)**, APC-Cy7 **(B)**, PerCP **(C)** and FITC **(D)**. The negative and positive populations were distinguished by the cell population count and mean for each fluorochrome where they were higher in positive population.

Supplementary Figure 4 Gating strategy for analysis of CD3^+^CD4^+^ Th cells and IL-17RA^+^ or IL-23R^+^ Th cells subset. Lymphocytes from PBMCs were gated according to forward-scatter (FSC) and side-scatter (SSC) **(A)**, and further gated for CD3^+^CD4^+^ Th cells population, as well as IL-17RA^+^ or IL-23R^+^ Th cells subset **(B)**. Numbers in each histogram represent positive populations; IL-17RA and IL-23R were labeled with PerCP and FITC, respectively.

Supplementary Figure 5 Proportion of IL-17RA^+^ **(A)** and IL-23R^+^ **(B)** Th cells subsets in SLE patients and healthy controls within Malay subjects.

## References

[1] Kasper IR Apostolidis SA Sharabi A Tsokos GC. Empowering Regulatory T Cells in Autoimmunity. Trends Mol Med (2016) 22:784–97. 10.1016/j.molmed.2016.07.003 PMC5003773 27461103

[2] Katsuyama T Tsokos GC Moulton VR. Aberrant T Cell Signaling and Subsets in Systemic Lupus Erythematosus. Front Immunol (2018) 9:1088. 10.3389/fimmu.2018.01088 29868033 PMC5967272

[3] Suarez-Fueyo A Bradley SJ Klatzmann D Tsokos GC. T Cells and Autoimmune Kidney Disease. Nat Rev Nephrol (2017) 13:329–43. 10.1038/nrneph.2017.34 28287110

[4] Sharabi A Tsokos GC. T Cell Metabolism: New Insights in Systemic Lupus Erythematosus Pathogenesis and Therapy. Nat Rev Rheumatol (2020) 16:100–12. 10.1038/s41584-019-0356-x 31949287

[5] Mohd Shukri ND Farah Izati A Wan Ghazali WS Che Hussin CM Wong KK. CD3(+)CD4(+)gp130(+) T Cells Are Associated With Worse Disease Activity in Systemic Lupus Erythematosus Patients. Front Immunol (2021) 12:675250. 10.3389/fimmu.2021.675250 34149710 PMC8213373

[6] Guerra ES Lee CK Specht CA Yadav B Huang H Akalin A. Central Role of IL-23 and IL-17 Producing Eosinophils as Immunomodulatory Effector Cells in Acute Pulmonary Aspergillosis and Allergic Asthma. PLoS Pathog (2017) 13:e1006175. 10.1371/journal.ppat.1006175 28095479 PMC5271415

[7] Chen Z Bozec A Ramming A Schett G. Anti-Inflammatory and Immune-Regulatory Cytokines in Rheumatoid Arthritis. Nat Rev Rheumatol (2019) 15:9–17. 10.1038/s41584-018-0109-2 30341437

[8] Huang X Hua J Shen N Chen S. Dysregulated Expression of Interleukin-23 and Interleukin-12 Subunits in Systemic Lupus Erythematosus Patients. Mod Rheumatol (2007) 17:220–3. 10.1007/s10165-007-0568-9 17564777

[9] Riol-Blanco L Lazarevic V Awasthi A Mitsdoerffer M Wilson BS Croxford A. IL-23 Receptor Regulates Unconventional IL-17-Producing T Cells That Control Bacterial Infections. J Immunol (2010) 184:1710–20. 10.4049/jimmunol.0902796 PMC2829977 20083652

[10] Ronnblom L Eloranta ML Alm GV. The Type I Interferon System in Systemic Lupus Erythematosus. Arthritis Rheum (2006) 54:408–20. 10.1002/art.21571 16447217

[11] McKenzie BS Kastelein RA Cua DJ. Understanding the IL-23-IL-17 Immune Pathway. Trends Immunol (2006) 27:17–23. 10.1016/j.it.2005.10.003 16290228

[12] Wong CK Lit LC Tam LS Li EK Wong PT Lam CW. Hyperproduction of IL-23 and IL-17 in Patients With Systemic Lupus Erythematosus: Implications for Th17-Mediated Inflammation in Auto-Immunity. Clin Immunol (2008) 127:385–93. 10.1016/j.clim.2008.01.019 18373953

[13] Farah Izati A Wong KK Che Maraina CH. IL-23/IL-17 Axis in the Pathogenesis and Treatment of Systemic Lupus Erythematosus and Rheumatoid Arthritis. Malays J Pathol (2020) 42:333–47. 33361714

[14] Ho AW Gaffen SL. IL-17RC: A Partner in IL-17 Signaling and Beyond. Semin Immunopathol (2010) 32:33–42. 10.1007/s00281-009-0185-0 20012905 PMC2837117

[15] Kastelein RA Hunter CA Cua DJ. Discovery and Biology of IL-23 and IL-27: Related But Functionally Distinct Regulators of Inflammation. Annu Rev Immunol (2007) 25:221–42. 10.1146/annurev.immunol.22.012703.104758 17291186

[16] Kyttaris VC Zhang Z Kuchroo VK Oukka M Tsokos GC. Cutting Edge: IL-23 Receptor Deficiency Prevents the Development of Lupus Nephritis in C57BL/6-Lpr/Lpr Mice. J Immunol (2010) 184:4605–9. 10.4049/jimmunol.0903595 PMC2926666 20308633

[17] Syahidatulamali CS Wan Syamimee WG Azwany YN Wong KK Che Maraina CH. Association of Anti-CLIC2 and Anti-HMGB1 Autoantibodies With Higher Disease Activity in Systemic Lupus Erythematosus Patients. J Postgrad Med (2017) 63:257–61. 10.4103/jpgm.JPGM_499_16 PMC5664871 28862243

[18] Nazri S Wong KK Hamid W. Pediatric Systemic Lupus Erythematosus. Retrospective Analysis of Clinico-Laboratory Parameters and Their Association With Systemic Lupus Erythematosus Disease Activity Index Score. Saudi Med J (2018) 39:627–31. 10.15537/smj.2018.6.22112 PMC6146216 29915860

[19] Parodis I Emamikia S Gomez A Gunnarsson I van Vollenhoven RF Chatzidionysiou K. Clinical SLEDAI-2K Zero may be a Pragmatic Outcome Measure in SLE Studies. Expert Opin Biol Ther (2019) 19:157–68. 10.1080/14712598.2019.1561856 30571926

[20] Jesus D Rodrigues M Matos A Henriques C Pereira da Silva JA Ines LS. Performance of SLEDAI-2K to Detect a Clinically Meaningful Change in SLE Disease Activity: A 36-Month Prospective Cohort Study of 334 Patients. Lupus (2019) 28:607–12. 10.1177/0961203319836717 30895904

[21] Vincent FB Northcott M Hoi A Mackay F Morand EF. Clinical Associations of Serum Interleukin-17 in Systemic Lupus Erythematosus. Arthritis Res Ther (2013) 15:R97. 10.1186/ar4277 23968496 PMC3979031

[22] Martin JC Baeten DL Josien R. Emerging Role of IL-17 and Th17 Cells in Systemic Lupus Erythematosus. Clin Immunol (2014) 154:1–12. 10.1016/j.clim.2014.05.004 24858580

[23] Mok MY Wu HJ Lo Y Lau CS. The Relation of Interleukin 17 (IL-17) and IL-23 to Th1/Th2 Cytokines and Disease Activity in Systemic Lupus Erythematosus. J Rheumatol (2010) 37:2046–52. 10.3899/jrheum.100293 20682672

[24] Zhao XF Pan HF Yuan H Zhang WH Li XP Wang GH. Increased Serum Interleukin 17 in Patients With Systemic Lupus Erythematosus. Mol Biol Rep (2010) 37:81–5. 10.1007/s11033-009-9533-3 19347604

[25] Cheng F Guo Z Xu H Yan D Li Q. Decreased Plasma IL22 Levels, But Not Increased IL17 and IL23 Levels, Correlate With Disease Activity in Patients With Systemic Lupus Erythematosus. Ann Rheum Dis (2009) 68:604–6. 10.1136/ard.2008.097089 19286907

[26] Nordin F Shaharir SS Abdul Wahab A Mustafar R Abdul Gafor AH Mohamed Said MS. Serum and Urine Interleukin-17A Levels as Biomarkers of Disease Activity in Systemic Lupus Erythematosus. Int J Rheum Dis (2019) 22:1419–26. 10.1111/1756-185X.13615 31179646

[27] Yang J Yang X Yang J Li M. Hydroxychloroquine Inhibits the Differentiation of Th17 Cells in Systemic Lupus Erythematosus. J Rheumatol (2018) 45:818–26. 10.3899/jrheum.170737 29545450

[28] Coquet JM Chakravarti S Kyparissoudis K McNab FW Pitt LA McKenzie BS. Diverse Cytokine Production by NKT Cell Subsets and Identification of an IL-17-Producing CD4-NK1.1- NKT Cell Population. Proc Natl Acad Sci USA (2008) 105:11287–92. 10.1073/pnas.0801631105 PMC2516267 18685112

[29] Tang C Chen S Qian H Huang W. Interleukin-23: As a Drug Target for Autoimmune Inflammatory Diseases. Immunology (2012) 135:112–24. 10.1111/j.1365-2567.2011.03522.x PMC3277713 22044352

[30] Chen K Kolls JK. Interluekin-17a (IL17A). Gene (2017) 614:8–14. 10.1016/j.gene.2017.01.016 28122268 PMC5394985

[31] Ma H Yuan Y Zhao L Ye Z Xu J Li M. Association of Gammadelta T Cell Compartment Size to Disease Activity and Response to Therapy in SLE. PLoS One (2016) 11:e0157772. 10.1371/journal.pone.0157772 27333282 PMC4917177

[32] Dai H He F Tsokos GC Kyttaris VC. IL-23 Limits the Production of IL-2 and Promotes Autoimmunity in Lupus. J Immunol (2017) 199:903–10. 10.4049/jimmunol.1700418 PMC5526729 28646040

[33] Shahin D El-Farahaty RM Houssen ME Machaly SA Sallam M ElSaid TO. Serum 25-OH Vitamin D Level in Treatment-Naive Systemic Lupus Erythematosus Patients: Relation to Disease Activity, IL-23 and IL-17. Lupus (2017) 26:917–26. 10.1177/0961203316682095 27927883

[34] Lopez P Rodriguez-Carrio J Caminal-Montero L Mozo L Suarez A pathogenic IFNalpha A. BLyS and IL-17 Axis in Systemic Lupus Erythematosus Patients. Sci Rep (2016) 6:20651. 10.1038/srep20651 26847824 PMC4742957

[35] Puwipirom H Hirankarn N Sodsai P Avihingsanon Y Wongpiyabovorn J Palaga T. Increased Interleukin-23 Receptor(+) T Cells in Peripheral Blood Mononuclear Cells of Patients With Systemic Lupus Erythematosus. Arthritis Res Ther (2010) 12:R215. 10.1186/ar3194 21110900 PMC3046525

[36] Petri M Orbai AM Alarcon GS Gordon C Merrill JT Fortin PR. Derivation and Validation of the Systemic Lupus International Collaborating Clinics Classification Criteria for Systemic Lupus Erythematosus. Arthritis Rheum (2012) 64:2677–86. 10.1002/art.34473 PMC3409311 22553077

[37] Hochberg MC. Updating the American College of Rheumatology Revised Criteria for the Classification of Systemic Lupus Erythematosus. Arthritis Rheum (1997) 40:1725. 10.1002/art.1780400928 9324032

[38] Gladman DD Ibanez D Urowitz MB. Systemic Lupus Erythematosus Disease Activity Index 2000. J Rheumatol (2002) 29:288–91. 11838846

[39] Chan EK Damoiseaux J Carballo OG Conrad K de Melo Cruvinel W Francescantonio PL. Report of the First International Consensus on Standardized Nomenclature of Antinuclear Antibody HEp-2 Cell Patterns 2014-2015. Front Immunol (2015) 6:412. 10.3389/fimmu.2015.00412 26347739 PMC4542633

[40] Kim HY. Statistical Notes for Clinical Researchers: Chi-Squared Test and Fisher's Exact Test. Restor Dent Endod (2017) 42:152–5. 10.5395/rde.2017.42.2.152 PMC5426219 28503482

[41] Isganaitis E Venditti S Matthews TJ Lerin C Demerath EW Fields DA. Maternal Obesity and the Human Milk Metabolome: Associations With Infant Body Composition and Postnatal Weight Gain. Am J Clin Nutr (2019) 110:111–20. 10.1093/ajcn/nqy334 PMC6599743 30968129

[42] Butler JM Maruska KP. The Mechanosensory Lateral Line System Mediates Activation of Socially-Relevant Brain Regions During Territorial Interactions. Front Behav Neurosci (2016) 10:93. 10.3389/fnbeh.2016.00093 27242462 PMC4865491

[43] Langrish CL Chen Y Blumenschein WM Mattson J Basham B Sedgwick JD. IL-23 Drives a Pathogenic T Cell Population That Induces Autoimmune Inflammation. J Exp Med (2005) 201:233–40. 10.1084/jem.20041257 PMC2212798 15657292

[44] Astry B Venkatesha SH Moudgil KD. Involvement of the IL-23/IL-17 Axis and the Th17/Treg Balance in the Pathogenesis and Control of Autoimmune Arthritis. Cytokine (2015) 74:54–61. 10.1016/j.cyto.2014.11.020 25595306 PMC4457562

[45] Bedoya SK Lam B Lau K Larkin J. 3rd, Th17 Cells in Immunity and Autoimmunity. Clin Dev Immunol (2013) 2013:986789. 10.1155/2013/986789 24454481 PMC3886602

[46] Lee PW Smith AJ Yang Y Selhorst AJ Liu Y Racke MK. IL-23R-Activated STAT3/STAT4 Is Essential for Th1/Th17-Mediated CNS Autoimmunity. JCI Insight (2017) 2:e91663. 10.1172/jci.insight.91663 PMC5621925 28878115

[47] Fischer K Przepiera-Bedzak H Sawicki M Walecka A Brzosko I Brzosko M. Serum Interleukin-23 in Polish Patients With Systemic Lupus Erythematosus: Association With Lupus Nephritis, Obesity, and Peripheral Vascular Disease. Mediators Inflamm (2017) 2017:9401432. 10.1155/2017/9401432 29430084 PMC5752988

[48] Abou Ghanima AT Elolemy GG Ganeb SS Abo Elazem AA Abdelgawad ER. Role of T Helper 17 Cells in the Pathogenesis of Systemic Lupus Erythematosus. Egypt J Immunol (2012) 19:25–33. 23885404

[49] Tsakonas E Joseph L Esdaile JM Choquette D Senecal JL Cividino A. A Long-Term Study of Hydroxychloroquine Withdrawal on Exacerbations in Systemic Lupus Erythematosus. The Canadian Hydroxychloroquine Study Group. Lupus (1998) 7:80–5. 10.1191/096120398678919778 9541091

[50] Fernandez M McGwin G Jr. Bertoli AM Calvo-Alen J Vila LM Reveille JD. Discontinuation Rate and Factors Predictive of the Use of Hydroxychloroquine in LUMINA, a Multiethnic US Cohort (LUMINA Xl). Lupus (2006) 15:700–4. 10.1177/0961203306072426 17120601

[51] Alarcon GS McGwin G Bertoli AM Fessler BJ Calvo-Alen J Bastian HM. Effect of Hydroxychloroquine on the Survival of Patients With Systemic Lupus Erythematosus: Data From LUMINA, a Multiethnic US Cohort (LUMINA L). Ann Rheum Dis (2007) 66:1168–72. 10.1136/ard.2006.068676 PMC1955128 17389655

[52] Fessler BJ Alarcon GS McGwin G Jr Roseman J Bastian HM Friedman AW. Systemic Lupus Erythematosus in Three Ethnic Groups: XVI. Association of Hydroxychloroquine Use With Reduced Risk of Damage Accrual. Arthritis Rheum (2005) 52:1473–80. 10.1002/art.21039 15880829

[53] Willis R Seif AM McGwin G Jr Martinez-Martinez LA Gonzalez EB Dang N. Effect of Hydroxychloroquine Treatment on Pro-Inflammatory Cytokines and Disease Activity in SLE Patients: Data From LUMINA (LXXV), a Multiethnic US Cohort. Lupus (2012) 21:830–5. 10.1177/0961203312437270 PMC3808832 22343096

[54] Garg S Unnithan R Hansen KE Costedoat-Chalumeau N Bartels CM. Clinical Significance of Monitoring Hydroxychloroquine Levels in Patients With Systemic Lupus Erythematosus: A Systematic Review and Meta-Analysis. Arthritis Care Res (Hoboken) (2021) 73:707–16. 10.1002/acr.24155 32004406

[55] Silva JC Mariz HA Rocha LF Jr. Oliveira PS Dantas AT Duarte AL. Hydroxychloroquine Decreases Th17-Related Cytokines in Systemic Lupus Erythematosus and Rheumatoid Arthritis Patients. Clinics (Sao Paulo) (2013) 68:766–71. 10.6061/clinics/2013(06)07 PMC3674253 23778483

[56] Abdel Galil SM Ezzeldin N El-Boshy ME. The Role of Serum IL-17 and IL-6 as Biomarkers of Disease Activity and Predictors of Remission in Patients With Lupus Nephritis. Cytokine (2015) 76:280–7. 10.1016/j.cyto.2015.05.007 26073684

[57] Raymond W Ostli-Eilertsen G Griffiths S Nossent J. IL-17A Levels in Systemic Lupus Erythematosus Associated With Inflammatory Markers and Lower Rates of Malignancy and Heart Damage: Evidence for a Dual Role. Eur J Rheumatol (2017) 4:29–35. 10.5152/eurjrheum.2017.16059 28293450 PMC5335884

[58] Du J Li Z Shi J Bi L. Associations Between Serum Interleukin-23 Levels and Clinical Characteristics in Patients With Systemic Lupus Erythematosus. J Int Med Res (2014) 42:1123–30. 10.1177/0300060513509130 24990863

[59] Crosslin KL Wiginton KL. The Impact of Race and Ethnicity on Disease Severity in Systemic Lupus Erythematosus. Ethn Dis (2009) 19:301–7. 19769013

[60] Cervera R Doria A Amoura Z Khamashta M Schneider M Guillemin F. Patterns of Systemic Lupus Erythematosus Expression in Europe. Autoimmun Rev (2014) 13:621–9. 10.1016/j.autrev.2013.11.007 24418306

[61] Kim SK Choe JY Lee SS. Self-Reported Physical Activity Is Associated With Lupus Nephritis in Systemic Lupus Erythematosus: Data From KORean Lupus Network (KORNET) Registry. Yonsei Med J (2018) 59:857–64. 10.3349/ymj.2018.59.7.857 PMC6082985 30091319

[62] DeQuattro K Trupin L Murphy LB Rush S Criswell LA Lanata CM. High Disease Severity Among Asians in a US Multiethnic Cohort of Individuals With Systemic Lupus Erythematosus. Arthritis Care Res (Hoboken) (2020). 10.1002/acr.24544 PMC8211905 33337580

[63] Frodlund M Wettero J Dahle C Dahlstrom O Skogh T Ronnelid J. Longitudinal Anti-Nuclear Antibody (ANA) Seroconversion in Systemic Lupus Erythematosus: A Prospective Study of Swedish Cases With Recent-Onset Disease. Clin Exp Immunol (2020) 199:245–54. 10.1111/cei.13402 PMC7008226 31778219

[64] Willems P De Langhe E Westhovens R Vanderschueren S Blockmans D Bossuyt X. Antinuclear Antibody as Entry Criterion for Classification of Systemic Lupus Erythematosus: Pitfalls and Opportunities. Ann Rheum Dis (2019) 78:e76. 10.1136/annrheumdis-2018-213821 29936439

[65] Wallace DJ Stohl W Furie RA Lisse JR McKay JD Merrill JT. Randomized, Double-Blind, Placebo-Controlled, Dose-Ranging Study of Belimumab in Patients With Active Systemic Lupus Erythematosus. Arthritis Rheum (2009) 61:1168–78. 10.1002/art.24699 PMC2758229 19714604

[66] Pisetsky DS Spencer DM Lipsky PE Rovin BH. Assay Variation in the Detection of Antinuclear Antibodies in the Sera of Patients With Established SLE. Ann Rheum Dis (2018) 77:911–3. 10.1136/annrheumdis-2017-212599 29440000

[67] Pisetsky DS Thompson DK Wajdula J Diehl A Sridharan S. Variability in Antinuclear Antibody Testing to Assess Patient Eligibility for Clinical Trials of Novel Treatments for Systemic Lupus Erythematosus. Arthritis Rheumatol (2019) 71:1534–8. 10.1002/art.40910 31385442

[68] Atamaniuk J Hsiao YY Mustak M Bernhard D Erlacher L Fodinger M. Analysing Cell-Free Plasma DNA and SLE Disease Activity. Eur J Clin Invest (2011) 41:579–83. 10.1111/j.1365-2362.2010.02435.x 21128939

[69] Conti F Ceccarelli F Perricone C Massaro L Marocchi E Miranda F. Systemic Lupus Erythematosus With and Without Anti-dsDNA Antibodies: Analysis From a Large Monocentric Cohort. Mediators Inflamm (2015) 2015:328078. 10.1155/2015/328078 26063969 PMC4438145

[70] Gheita TA Abaza NM Hammam N Mohamed AAA El G II Eissa AH. Anti-dsDNA Titre in Female Systemic Lupus Erythematosus Patients: Relation to Disease Manifestations, Damage and Antiphospholipid Antibodies. Lupus (2018) 27:1081–7. 10.1177/0961203318760209 29460701

[71] Rekvig OP. Anti-dsDNA Antibodies as a Classification Criterion and a Diagnostic Marker for Systemic Lupus Erythematosus: Critical Remarks. Clin Exp Immunol (2015) 179:5–10. 10.1111/cei.12296 24533624 PMC4260890

[72] Villalta D Bizzaro N Bassi N Zen M Gatto M Ghirardello A. Anti-dsDNA Antibody Isotypes in Systemic Lupus Erythematosus: IgA in Addition to IgG Anti-dsDNA Help to Identify Glomerulonephritis and Active Disease. PLoS One (2013) 8:e71458. 10.1371/journal.pone.0071458 23951169 PMC3741383

