# Supplementary material for: Increased IL-23R+ Th Cells Population Exhibits Higher SLEDAI-2K Scores in Systemic Lupus Erythematosus Patients
Source: Front Immunol. 2021 Aug 17;12:690908. doi: 10.3389/fimmu.2021.690908 (PMC8416093; doi:10.3389/fimmu.2021.690908)

Supplementary Figure 1

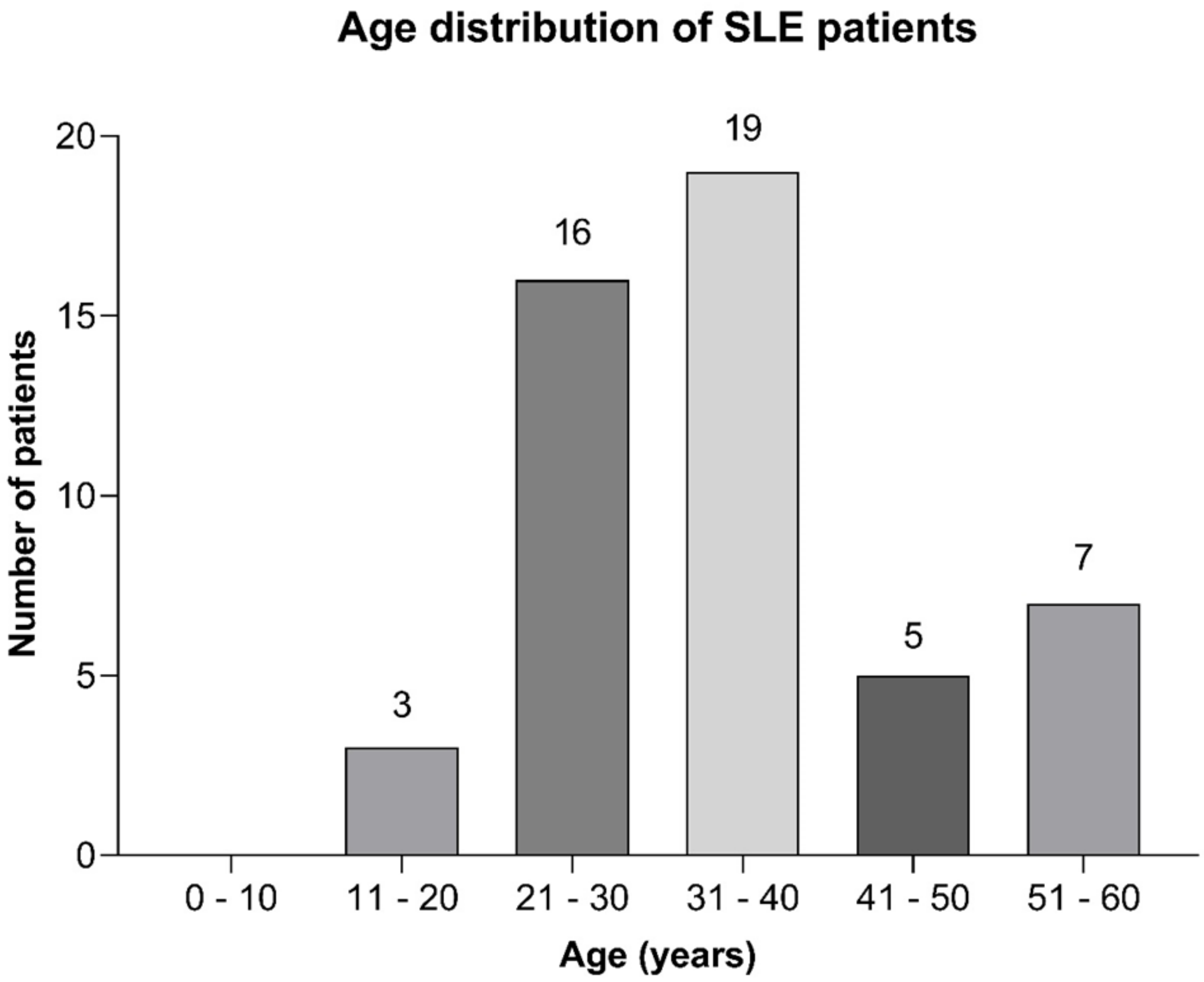

Supplementary Figure 2

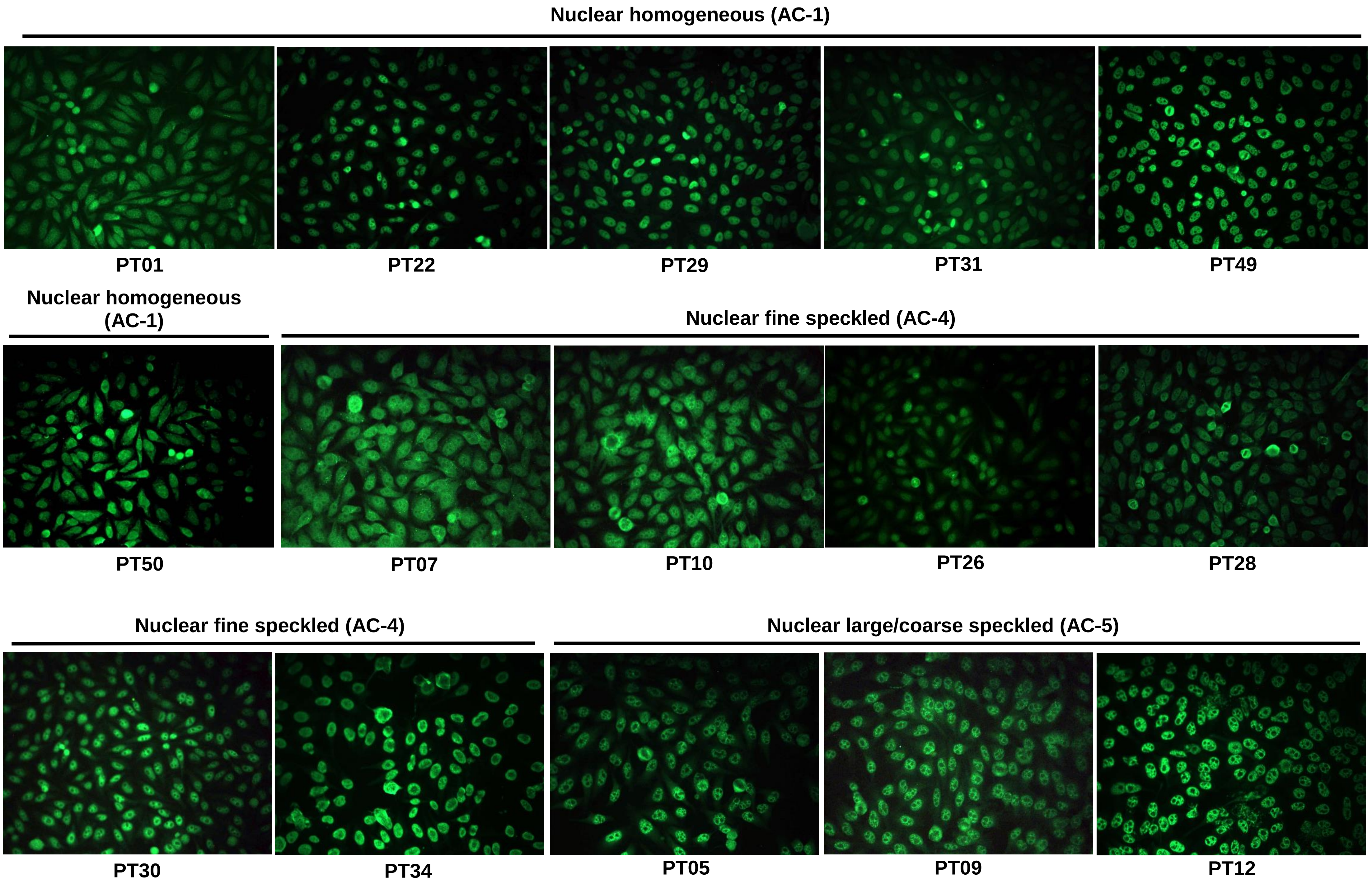

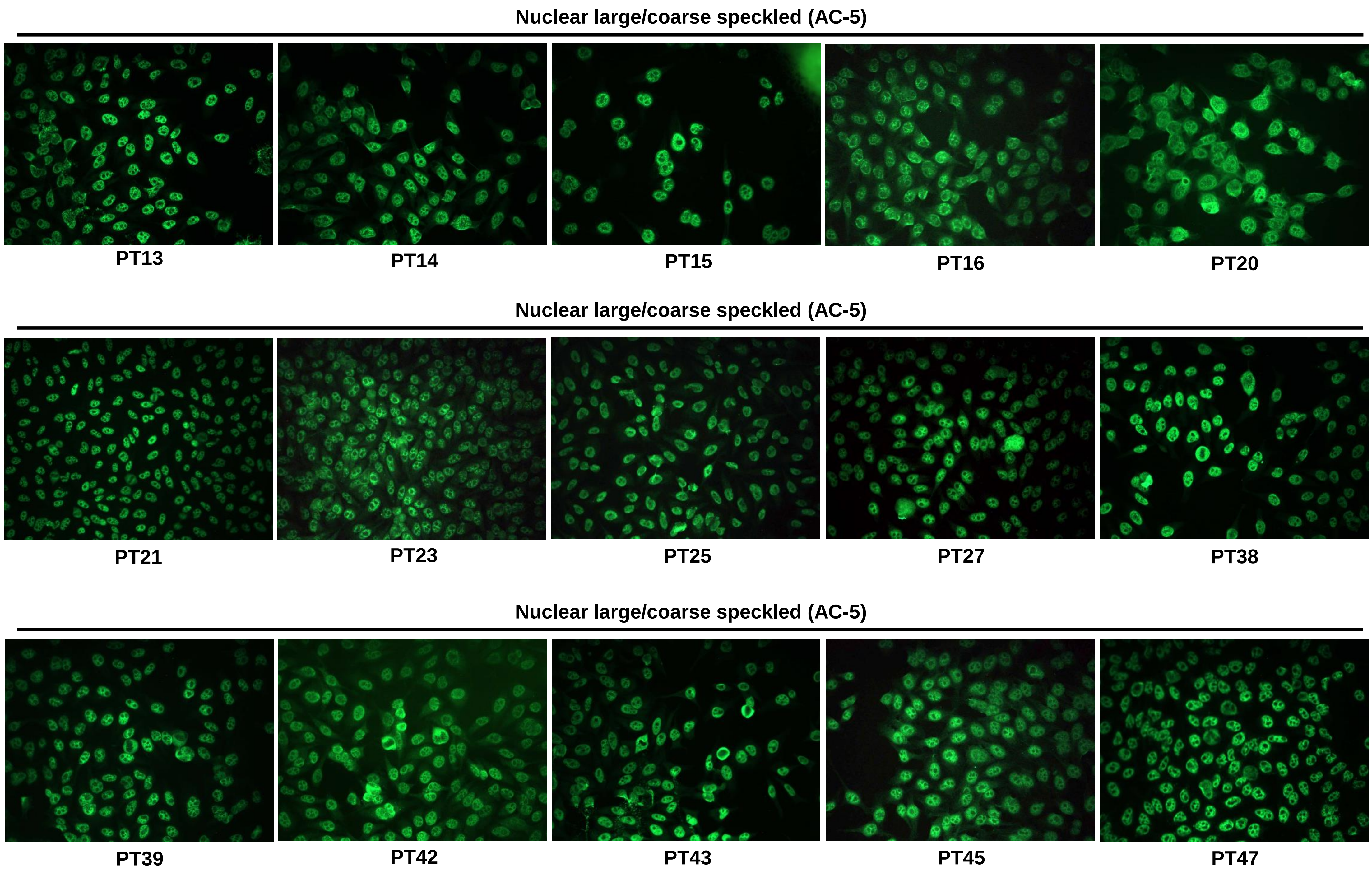
Nuclear large/coarse speckled (AC-5)
PT13
PT14
PT15
PT16
PT20
Nuclear large/coarse speckled (AC-5)
PT21
PT23
PT25
PT27
PT38
Nuclear large/coarse speckled (AC-5)
PT39
PT42
PT43
PT45
PT47

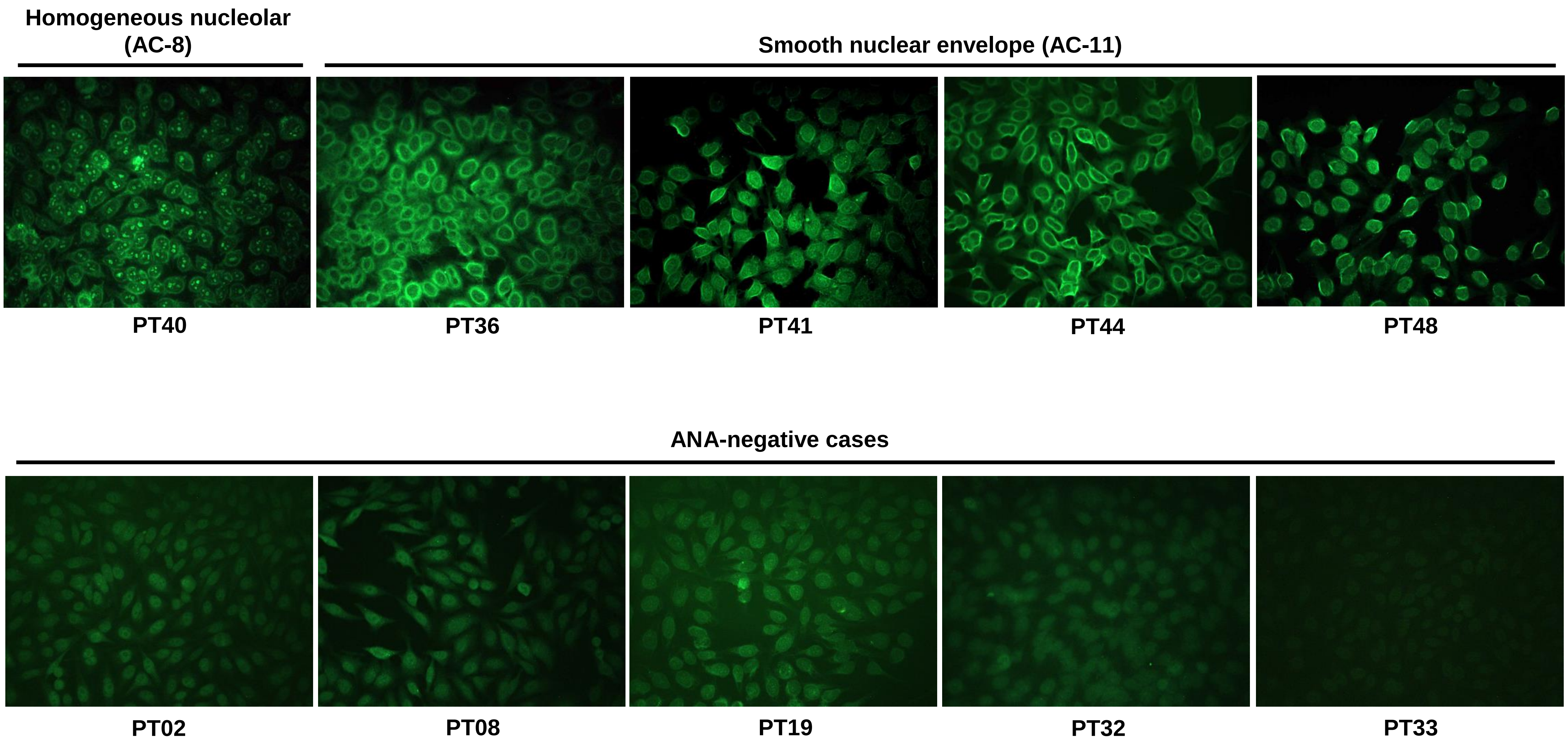
Homogeneous nucleolar (AC-8)
Smooth nuclear envelope (AC-11)
PT40
PT36
PT41
PT44
PT48
ANA-negative cases
PT02
PT08
PT19
PT32
PT33

Supplementary Figure 3

(A)

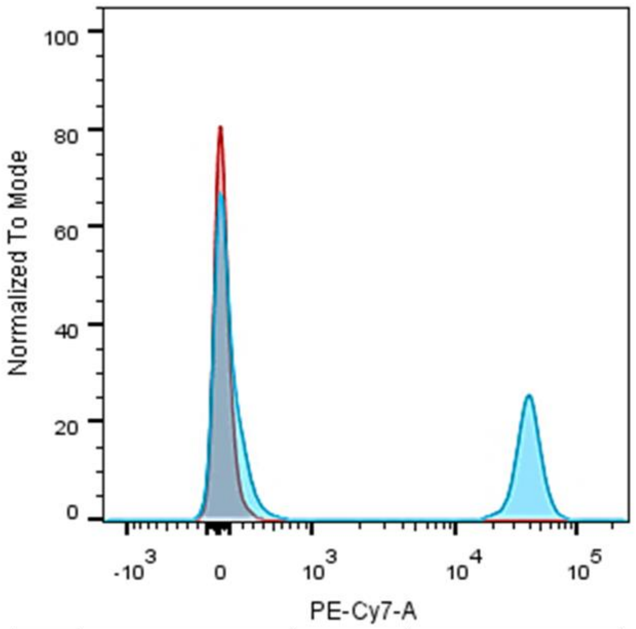

	Subset Name	Count	Mean: PE-Cy7
■	Isotype	307	14.0
■	PE-Cy7	371	2724

(B)

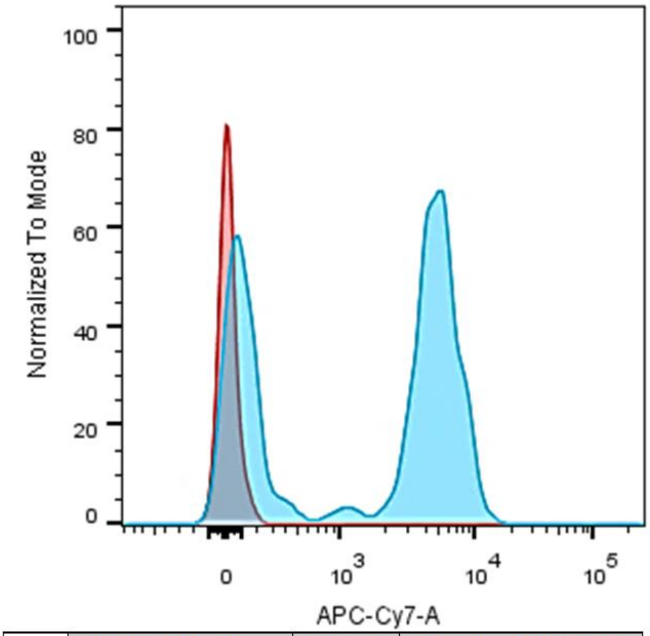

	Subset Name	Count	Mean: APC-Cy7
■	Isotype	374	4.56
■	APC-Cy7	380	3424

(C)

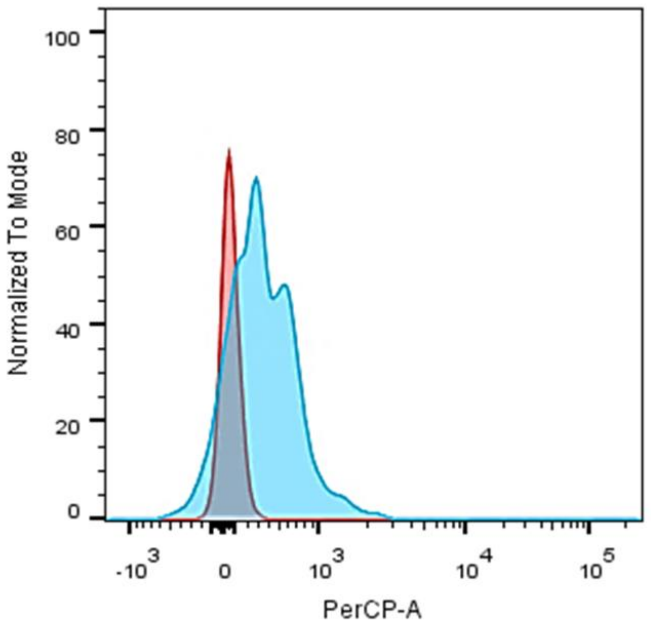

	Subset Name	Count	Mean: PerCP
■	Isotype	374	41.4
■	PerCP	383	388

(D)

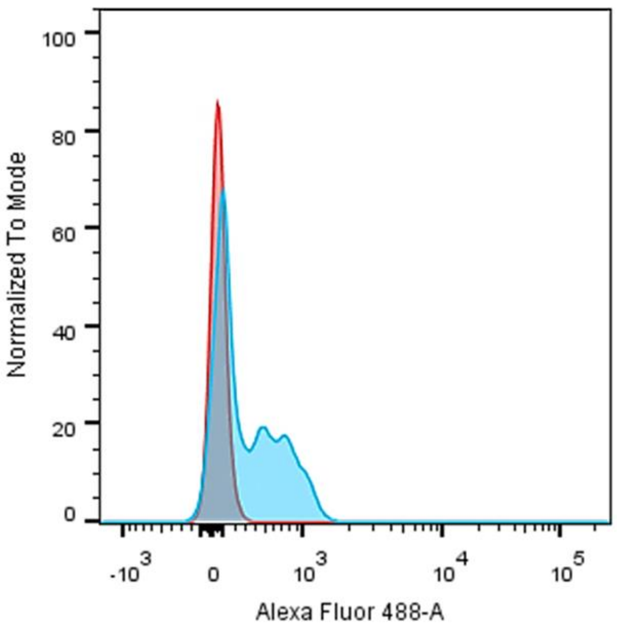

	Subset Name	Count	Mean: FITC
■	Isotype	371	42.5
■	FITC	483	354

Supplementary Figure 4

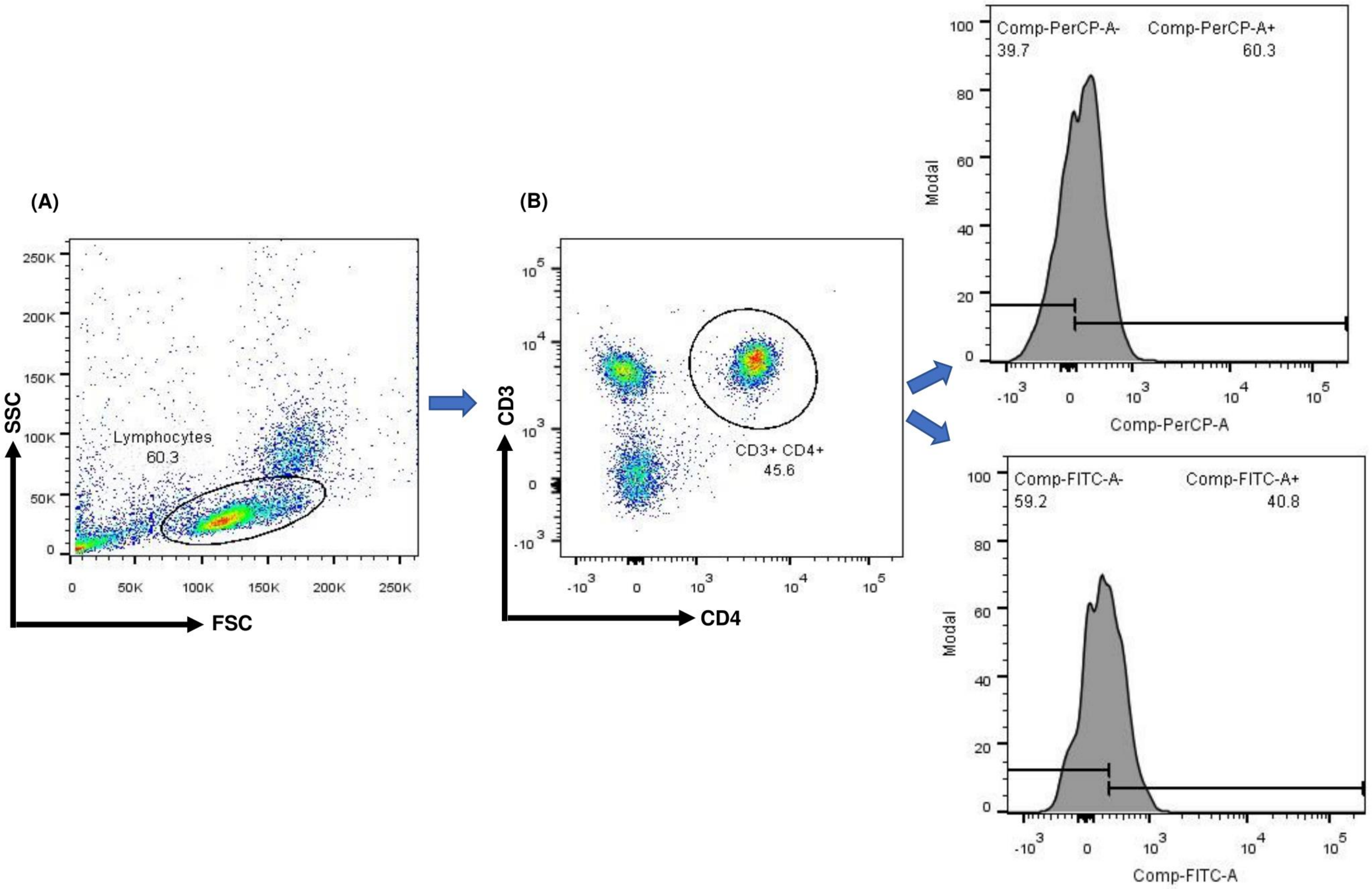

Supplementary Figure 5

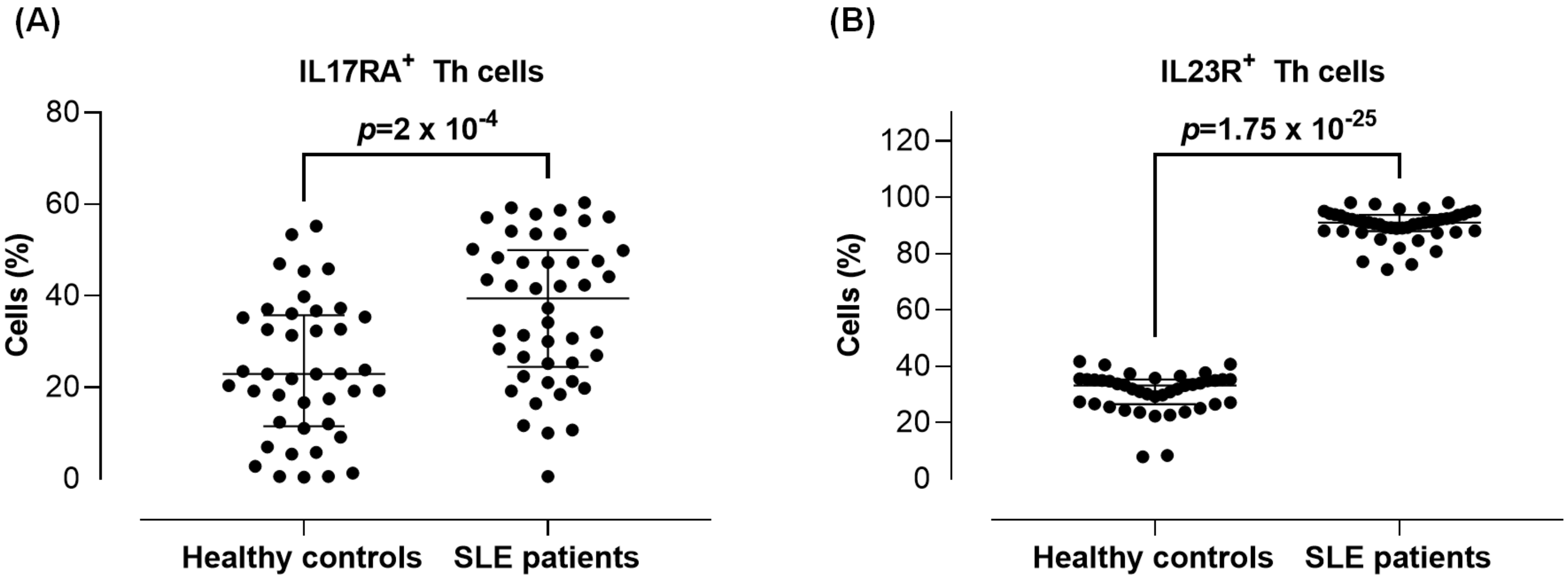

Supplement: Supplementary Figure 1 — Distribution of age in 50 SLE patients. [file DataSheet_2.pdf]
