# Supplementary material for: Increased IL-23R+ Th Cells Population Exhibits Higher SLEDAI-2K Scores in Systemic Lupus Erythematosus Patients
Source: Front Immunol. 2021 Aug 17;12:690908. doi: 10.3389/fimmu.2021.690908 (PMC8416093; doi:10.3389/fimmu.2021.690908)
Supplement: Supplementary file 2 [file DataSheet_1.pdf]

Supplementary Table 1. Inclusion and exclusion criteria of study subjects.

**Inclusion criteria**	**Exclusion criteria**
SLE patients<br>• Age between 18-60 years old.<br>• Adults SLE patients who fulfilled the 2012 Systemic Lupus International Collaborating Clinics (SLICC) criteria for SLE diagnosed from year 2012 onwards, or the 1997 Update of the 1982 American College of Rheumatology Revised Criteria for Classification of SLE for patients diagnosed before 2012.	*SLE patients*<br>• Drug-induced lupus patients.<br>• Pregnant patients.
Healthy controls<br>• Normal healthy volunteers without medical illness.<br>• Age between 18-60 years old.	*Healthy controls*<br>• Volunteers with an immunosuppressed condition such as diabetes mellitus, cancers and/or AIDS or HIV-positive.<br>• Pregnant patients.<br>• Have recent inflammation or infection 2 weeks prior to study.

Supplementary Table 2. ANA and anti-dsDNA test results of SLE patients prior to disease diagnosis and present results including CRP.

No.	Patient ID no.	Results leading to SLE diagnosis			Present results*			
		ANA	Anti-dsDNA	Year	ANA	Anti-dsDNA		CRP levels
1	PT01	POS	POS	2016	POS	POS	187.6	0
2	PT02	POS	N/A	2015	NEG	NEG	49	0
3	PT03	POS	N/A	2015	POS	POS	98.384	0
4	PT04	POS	N/A	2009	POS	POS	145.06	21
5	PT05	POS	POS	2010	POS	POS	115.6	6
6	PT06	POS	POS	2007	POS	POS	269.36	5
7	PT07	POS	POS	2017	POS	POS	126.2	0
8	PT08	POS	POS	2013	NEG	POS	106.3	0
9	PT09	POS	POS	2014	POS	NEG	19.573	0
10	PT10	POS	N/A	2009	POS	NEG	26.118	36
11	PT11	POS	N/A	2018	POS	NEG	29.26	0
12	PT12	POS	POS	2011	POS	POS	111.28	0
13	PT13	POS	POS	2017	POS	POS	87.125	0
14	PT14	POS	POS	2018	POS	POS	252.93	0
15	PT15	POS	POS	2010	POS	POS	174.58	10
16	PT16	POS	NEG	2018	POS	NEG	49	24
17	PT17	POS	POS	2018	POS	POS	382.99	0
18	PT18	POS	POS	2011	POS	POS	230.8	0
19	PT19	POS	N/A	2015	NEG	NEG	27.559	11
20	PT20	POS	POS	2018	POS	POS	53.676	0
21	PT21	POS	POS	2019	POS	POS	308.83	0
22	PT22	POS	NEG	2016	POS	NEG	49	9
23	PT23	POS	POS	2015	POS	POS	377.82	0
24	PT24	POS	NEG	2014	POS	NEG	6.1539	0
25	PT25	POS	NEG	2015	POS	NEG	49	8

No.	Patient ID no.	Results leading to SLE diagnosis			Present results*			
		ANA	Anti-dsDNA	Year	ANA	Anti-dsDNA		CRP levels
26	PT26	POS	POS	2014	POS	POS	140.27	0
27	PT27	POS	POS	2017	POS	POS	113.51	7
28	PT28	POS	POS	2009	POS	POS	399.58	8
29	PT29	POS	NEG	2006	POS	POS	54.436	7
30	PT30	POS	POS	2010	POS	POS	236.36	6
31	PT31	POS	POS	2017	POS	NEG	49	0
32	PT32	POS	POS	2017	NEG	POS	220.74	0
33	PT33	POS	POS	2009	NEG	POS	171.92	8
34	PT34	POS	POS	2010	POS	NEG	11.965	7
35	PT35	POS	POS	2018	POS	NEG	8.1411	7
36	PT36	POS	POS	2012	POS	POS	299.99	0
37	PT37	POS	NEG	2013	POS	NEG	21.078	0
38	PT38	POS	POS	2009	POS	POS	147.35	9
39	PT39	POS	POS	2010	POS	POS	401	5
40	PT40	POS	N/A	2017	POS	POS	401	0
41	PT41	POS	POS	2011	POS	POS	401	0
42	PT42	POS	NEG	2015	POS	POS	101.87	7
43	PT43	POS	POS	2015	POS	POS	200.97	20
44	PT44	POS	POS	2011	POS	POS	107.32	0
45	PT45	POS	POS	2016	POS	POS	154.19	0
46	PT46	POS	POS	2012	POS	POS	65.013	0
47	PT47	POS	POS	2018	POS	POS	303.81	0
48	PT48	POS	POS	2019	POS	POS	401	0
49	PT49	POS	POS	2007	POS	NEG	48.578	7
50	PT50	POS	POS	2018	POS	POS	401	6

*Present results denote the time of the current study conducted (November 2018 – May 2019); Notes: Previous page displays SLE patients with current ANA negative, and this page displays SLE patients with current ANA positive.

Supplementary Table 3. ANA pattern interpretation for SLE patients (n=50).

ID	ANA pattern	ICAP code	Interpretation
PT01	Nuclear homogeneous	AC-1	Homogenous staining of nucleoplasm.
PT02	Negative	N/A	Negative.
PT03	Nuclear fine speckled	AC-4	Fine speckles in nucleoplasm with unstained nucleoli. Chromatin mass of mitotic cells not stained.
PT04	Homogeneous nucleolar	AC-8	Strong and homogenous staining of nucleoli.
PT05	Nuclear large/coarse speckled	AC-5	Coarse speckles across all nucleoplasm and nucleoli are unstained. Chromatin of mitotic cells not stained.
PT06	Nuclear homogeneous	AC-1	Homogenous staining of nucleoplasm. Intense staining of chromatin mass of metaphase cells.
PT07	Nuclear fine speckled	AC-4	Fine speckles across vast majority of cells' nucleoplasm with moderate intensity.<br>Chromatin of mitotic cells not stained.
PT08	Negative	N/A	Negative.
PT09	Nuclear large/coarse speckled	AC-5	Coarse speckles across all nucleoplasm and nucleoli are unstained. Chromatin of mitotic cells not stained.
PT10	Nuclear fine speckled	AC-4	Fine speckles in nucleoplasm with unstained nucleoli. Chromatin of mitotic cells not stained.
PT11	Centromere	AC-3	Several discrete coarse speckles in interphase cells.
PT12	Nuclear large/coarse speckled	AC-5	Coarse speckles across all nucleoplasm and nucleoli are unstained.
PT13	Nuclear large/coarse speckled	AC-5	Coarse speckles across all nucleoplasm and nucleoli are unstained. Chromatin of mitotic cells not stained.
PT14	Nuclear large/coarse speckled	AC-5	Coarse speckles across all nucleoplasm and nucleoli are unstained.
PT15	Nuclear large/coarse speckled	AC-5	Coarse speckles across all nucleoplasm and nucleoli are unstained. Chromatin of mitotic cells not stained.
PT16	Nuclear large/coarse speckled	AC-5	Coarse speckles across all nucleoplasm and nucleoli are unstained. Chromatin of mitotic cells not stained.
PT17	Smooth nuclear envelope	AC-11	Strong staining of nuclear outer rims of interphase cells and accentuation at the points where adjacent cells touch each other.
PT18	Cytoplasmic dense fine speckled	AC-19	Cloudy appearance with near homogeneous staining of cytoplasm.
PT19	Negative	N/A	Negative.
PT20	Nuclear large/coarse speckled	AC-5	Coarse speckles across all nucleoplasm and nucleoli are unstained. Chromatin of mitotic cells not stained.
PT21	Nuclear large/coarse speckled	AC-5	Coarse speckles across all nucleoplasm and nucleoli are unstained. Chromatin of mitotic cells not stained.
PT22	Nuclear homogeneous	AC-1	Homogenous staining of nucleoplasm (nucleoli no stained) and strong staining of chromatin mass of mitotic cells.
PT23	Nuclear large/coarse speckled	AC-5	Coarse speckles across all nucleoplasm and nucleoli are unstained. Chromatin of mitotic cells not stained.
PT24	Nuclear dense fine speckled	AC-2	Fine speckled pattern and strong staining of chromatin of metaphase cells.
PT25	Nuclear large/coarse speckled	AC-5	Coarse speckles across all nucleoplasm and nucleoli are unstained.

ID	ANA pattern	ICAP code	Interpretation
PT26	Nuclear fine speckled	AC-4	Fine speckles across vast majority of cells' nucleoplasm with acceptable intensity. Chromatin of mitotic cells not stained.
PT27	Nuclear large/coarse speckled	AC-5	Coarse speckles across all nucleoplasm and nucleoli are unstained.
PT28	Nuclear fine speckled	AC-4	Fine speckles in nucleoplasm with unstained nucleoli. Chromatin mass of mitotic cells not stained.
PT29	Nuclear homogeneous	AC-1	Homogenous staining of nucleoplasm (nucleoli no stained) and strong staining of chromatin of metaphase cells.
PT30	Nuclear fine speckled	AC-4	Fine speckles in nucleoplasm with unstained nucleoli.
PT31	Nuclear homogeneous	AC-1	Homogenous staining of nucleoplasm. Intense staining of chromatin mass of mitotic cells.
PT32	Negative	N/A	Negative.
PT33	Negative	N/A	Negative.
PT34	Nuclear fine speckled	AC-4	Fine speckles in nucleoplasm with mixture of stained and unstained nucleoli.
PT35	Nuclear large/coarse speckled	AC-5	Coarse speckles across all nucleoplasm and nucleoli are unstained. Chromatin of mitotic cells not stained.
PT36	Smooth nuclear envelope	AC-11	Strong staining of nuclear outer rims of interphase cells.
PT37	PCNA-like	AC-13	Speckled nucleoplasmic staining with heterogeneous intensity and size of speckles. Other interphase cells are negative.
PT38	Nuclear large/coarse speckled	AC-5	Coarse speckles across all nucleoplasm and nucleoli are unstained. Chromatin of mitotic cells not stained.
PT39	Nuclear large/coarse speckled	AC-5	Coarse speckles across all nucleoplasm and nucleoli are unstained. Chromatin of mitotic cells not stained.
PT40	Homogeneous nucleolar	AC-8	Homogenous intensity of nucleoli.
PT41	Smooth nuclear envelope	AC-11	Strong staining of nuclear outer rims of interphase cells.
PT42	Nuclear large/coarse speckled	AC-5	Coarse speckles across all nucleoplasm and nucleoli are unstained. Chromatin of mitotic cells not stained.
PT43	Nuclear large/coarse speckled	AC-5	Coarse speckles across all nucleoplasm and nucleoli are unstained. Chromatin of mitotic cells not stained.
PT44	Smooth nuclear envelope	AC-11	Strong staining of nuclear outer rims of interphase cells.
PT45	Nuclear large/coarse speckled	AC-5	Coarse speckles across all nucleoplasm and nucleoli are unstained.
PT46	NuMA-like	AC-26	Strong staining of spindle fibers. Interphase cells with weak speckled staining.
PT47	Nuclear large/coarse speckled	AC-5	Coarse speckles across all nucleoplasm and nucleoli are unstained.
PT48	Smooth nuclear envelope	AC-11	Strong staining of nuclear outer rims of interphase cells.
PT49	Nuclear homogeneous	AC-1	Homogenous staining of nucleoplasm (nucleoli no stained) and strong staining of chromatin mass of mitotic cells.
PT50	Nuclear homogeneous	AC-1	Homogenous staining of nucleoplasm.

Supplementary Table 4. Percentage of SLE patients demonstrating different ANA patterns in published literature according to ICAP nomenclature.

ANA pattern (ICAP code)	SLE patients with the ANA pattern, n (%)	Notes	References
AC-1	25 (46)	54 newly diagnosed SLE patients (<6 months diagnosed)	Frodlund M, Wettero J, Dahle C, Dahlstrom O, Skogh T, Ronnelid J, et al., Longitudinal anti-nuclear antibody (ANA) seroconversion in systemic lupus erythematosus: a prospective study of Swedish cases with recent-onset disease. *Clin Exp Immunol* (2020) 199:245-54. doi: 10.1111/cei.13402
AC-4	15 (27)		
AC-5	2 (4)		
AC-6	1 (2)		
AC-8	2 (4)		
AC-1/4	6 (11)		
AC-1/8-10	3 (6)		
AC-1	31 (23.6)	131/147 (89%) SLE patients were ANA positive	Wei Q, Jiang Y, Xiao M, Zhang X, Qi J, Xie J, et al., Comparison of chemiluminescence microparticle immunoassay, indirect immunofluorescence assay, linear immunoassay and multiple microbead immunoassay detecting autoantibodies in systemic lupus erythematosus. *Scand J Immunol* (2020) 91:e12849. doi: 10.1111/sji.12849
AC-4	52 (39.7)		
AC-5	32 (24.4)		
AC-8	3 (2.3)		
AC-9	4 (3.1)		
AC-19	1 (0.8)		
AC-7/AC-20	1 (0.8)		
AC-9/AC-19	1 (0.8)		
AC-1	415 (20.4)	1,844/2,034 (90.6%) SLE patients were ANA positive<br><br>This study was conducted to compare ANA positivity rate, titers and patients with SARD (SLE, RA, pSS, SSc, and MCTD).	Wei Q, Jiang Y, Xie J, Lv Q, Xie Y, Tu L, et al., Analysis of antinuclear antibody titers and patterns by using HEp-2 and primate liver tissue substrate indirect immunofluorescence assay in patients with systemic autoimmune rheumatic diseases. *J Clin Lab Anal* (2020) 34:e23546. doi: 10.1002/jcla.23546
AC-2	5 (0.2)		
AC-3	23 (0.1)		
AC-4	617 (30.3)		
AC-5	536 (26.4)		
AC-6	10 (0.5)		
AC-8/9	50 (2.6)		
AC-11/12	9 (0.4)		
AC-15	3 (0.2)		

AC-19	75 (3.7)		
AC-21	13 (0.6)		
Mixed	78 (3.8)		
Other	10 (0.5)		

SARD; systemic autoimmune rheumatic diseases, pSS; primary Sjogren’s syndrome, SSc; systemic sclerosis, MCTD; mixed connective tissue disease.

Supplementary Table 5. Association of serum IL-17 and IL-23 levels, and IL-17RA$^+$ or IL-23R$^+$ Th cells population with immunological parameters in SLE patients (n=50).

Parameters	ANA		*p*-value	Anti-dsDNA		*p*-value	CRP			*p*-value
	Negative, n=5	Positive, n=45		Negative, n=14	Positive, n=36		Normal	Mildly elevated	Elevated	
**IL-17 (pg/ml) (median; IQR)**	2.68 (0.43-5.05)	4.75 (2.06-5.26)	0.363[a]	2.78 (0.66-5.07)	4.96 (1.99-5.28)	0.177[a]	4.46 (1.61-5.25)	5.15 (1.70-5.39)	3.58 (2.00-5.15)	0.584[c]
**IL-23 (pg/ml) (median; IQR)**	36.10 (14.86-161.3)	22.39 (16.94-46.61)	0.340[a]	20.11 (15.76-27.70)	23.79 (18.25-56.50)	0.207[a]	24.31 (20.40-53.41)	18.27 (15.19-42.28)	22.34 (13.07-150.60)	0.217[c]
**IL-17RA$^+$ Th cells (%) (median; IQR)**	32.0 (17.50-49.25)	41.60 (26.0-50.05)	0.532[a]	37.40 (26.90-54.68)	39.45 (23.10-48.13)	0.551[b]	39.45 (23.10-48.05)	32.50 (27.35-54.73)	46.20 (35.48-54.23)	0.545[c]
**IL-23R$^+$ Th cells (%) (median; IQR)**	89.10 (81.20-94.60)	91.0 (87.95-93.70)	0.576[a]	90.95 (88.98-92.93)	87.53 (90.90-93.75)	0.673[a]	91.15 (88.93-94.58)	89.60 (87.53-93.15)	91.20 (88.0-92.18)	0.462[c]

[a]Mann-Whitney test; [b]Unpaired t-test; [c]Kruskal-Wallis test.

Supplementary Table 6. Correction for multiple comparisons in healthy controls (HCs; n=50) or SLE patients (n=50) with the Benjamini-Hochberg (BH) procedures and false discovery rate (FDR) of 0.25. Significance is in bold.

**No.**	**Comparison**	***p*-value**	**BH-corrected *p*-value**	**BH-corrected significance**
1	IL-17 vs IL-17RA$^+$ Th cells in HCs	0.130	0.584	Not significant
2	IL-23 vs IL-17RA$^+$ Th cells in HCs	0.275	0.707	Not significant
3	IL-17 vs IL-23R$^+$ Th cells in HCs	0.599	0.762	Not significant
4	IL-23 vs IL-23R$^+$ Th cells in HCs	0.809	0.857	Not significant
5	IL-17 vs IL-17RA$^+$ Th cells in SLE	**0.038**	0.252	Not significant
6	IL-23 vs IL-17RA$^+$ Th cells in SLE	0.681	0.766	Not significant
7	IL-17 vs IL-23R$^+$ Th cells in SLE	0.437	0.762	Not significant
8	IL-23 vs IL-23R$^+$ Th cells in SLE	0.884	0.909	Not significant
9	IL-17 vs SLEDAI-2K in SLE	0.508	0.762	Not significant
10	IL-23 vs SLEDAI-2K in SLE	0.466	0.762	Not significant
11	IL-17RA$^+$ Th cells vs SLEDAI-2K in SLE	0.743	0.811	Not significant
12	IL-23R$^+$ Th cells vs SLEDAI-2K in SLE	**0.017**	**0.204**	**Significant**
13	IL-17 vs ANA in SLE	0.363	0.762	Not significant
14	IL-23 vs ANA in SLE	0.340	0.762	Not significant
15	IL-17RA vs ANA in SLE	0.532	0.762	Not significant
16	IL-23R vs ANA in SLE	0.576	0.762	Not significant
17	IL-17 vs anti-dsDNA in SLE	0.177	0.601	Not significant
18	IL-23 vs anti-dsDNA in SLE	0.207	0.601	Not significant
19	IL-17RA vs anti-dsDNA in SLE	0.551	0.762	Not significant
20	IL-23R vs anti-dsDNA in SLE	0.673	0.766	Not significant
21	IL-17 vs CRP state in SLE	0.584	0.762	Not significant
22	IL-23 vs CRP state in SLE	0.217	0.601	Not significant
23	IL-17RA vs CRP state in SLE	0.545	0.762	Not significant
24	IL-23R vs CRP state in SLE	0.462	0.762	Not significant

No.	Comparison	***p*-value**	**BH-corrected *p*-value**	**BH-corrected significance**
25	Age vs SLEDAI-2K	**0.042**	0.252	Not significant
26	Ethnicity vs SLEDAI-2K	0.614	0.762	Not significant
27	History of autoimmune disease vs SLEDAI-2K	0.191	0.601	Not significant
28	Prednisolone vs SLEDAI-2K	**0.009**	**0.162**	**Significant**
29	Immunosuppressant vs SLEDAI-2K	0.441	0.762	Not significant
30	ANA vs SLEDAI-2K	0.651	0.766	Not significant
31	Anti-dsDNA vs SLEDAI-2K	0.324	0.762	Not significant
32	CRP vs SLEDAI-2K	0.999	0.999	Not significant
33	Multivariate analysis: Age (and IL-23R$^+$ Th cells) vs SLEDAI-2K	0.111	0.571	Not significant
34	Multivariate analysis: IL-23R$^+$ Th cells (and age) vs SLEDAI-2K	0.146	0.584	Not significant
35	Multivariate: Prednisolone (and IL-23R$^+$ Th cells) vs SLEDAI-2K	**0.006**	**0.162**	**Significant**
36	Multivariate: IL-23R$^+$ Th cells (and prednisolone) vs SLEDAI-2K	**0.027**	**0.243**	**Significant**
